# Development and validation of a high throughput SARS-CoV-2 whole genome sequencing workflow in a clinical laboratory

**DOI:** 10.1038/s41598-022-06091-0

**Published:** 2022-02-08

**Authors:** Sun Hee Rosenthal, Anna Gerasimova, Rolando Ruiz-Vega, Kayla Livingston, Ron M. Kagan, Yan Liu, Ben Anderson, Renius Owen, Laurence Bernstein, Alla Smolgovsky, Dong Xu, Rebecca Chen, Andrew Grupe, Pranoot Tanpaiboon, Felicitas Lacbawan

**Affiliations:** Quest Diagnostics, San Juan Capistrano, CA 92675 USA

**Keywords:** Infectious-disease diagnostics, Whole genome amplification

## Abstract

Monitoring new mutations in SARS-CoV-2 provides crucial information for identifying diagnostic and therapeutic targets and important insights to achieve a more effective COVID-19 control strategy. Next generation sequencing (NGS) technologies have been widely used for whole genome sequencing (WGS) of SARS-CoV-2. While various NGS methods have been reported, one chief limitation has been the complexity of the workflow, limiting the scalability. Here, we overcome this limitation by designing a laboratory workflow optimized for high-throughput studies. The workflow utilizes modified ARTIC network v3 primers for SARS-CoV-2 whole genome amplification. NGS libraries were prepared by a 2-step PCR method, similar to a previously reported tailed PCR method, with further optimizations to improve amplicon balance, to minimize amplicon dropout for viral genomes harboring primer-binding site mutation(s), and to integrate robotic liquid handlers. Validation studies demonstrated that the optimized workflow can process up to 2688 samples in a single sequencing run without compromising sensitivity and accuracy and with fewer amplicon dropout events compared to the standard ARTIC protocol. We additionally report results for over 65,000 SARS-CoV-2 whole genome sequences from clinical specimens collected in the United States between January and September of 2021, as part of an ongoing national genomics surveillance effort.

## Introduction

Coronavirus disease 2019 (COVID-19), caused by the severe acute respiratory syndrome coronavirus 2 (SARS-CoV-2), emerged in the Chinese province of Wuhan in November 2019^1,2^ and was declared a global pandemic by the World Health Organization after its rapid spread around the world (https://www.who.int/emergencies/diseases/novel-coronavirus-2019). Genomic surveillance has been employed to track the evolution of SARS-CoV-2 over the course of the pandemic, including the emergence of new variants that may affect viral transmissibility, infectivity, immune evasion, and vaccine efficacy^3–5^. It further informs public health decisions by facilitating the tracking of SARS-CoV-2 transmission, outbreaks, and individual contact tracing, and has been used to help trace the origin of the pandemic^6–8^. As of Sept 2021, more than three million SARS-CoV-2 genomic sequences worldwide have been deposited and made publicly available in the global initiative on sharing all influenza data (GISAID) database (https://www.gisaid.org/).

The initial SARS-CoV-2 genome sequence was obtained through a metagenomic approach and confirmed by Sanger sequencing and PCR^2,9,10^. As the pandemic progressed, multiple NGS approaches have been utilized for SARS-CoV-2 sequencing, including shotgun metagenomics, hybrid capture enrichment, and amplicon-based sequencing^11–14^. Shotgun sequencing requires no prior knowledge of the targeted viral genome^15^ but is limited by requirements for a high viral load and a higher sequencing depth. Hybridization approaches target genomic regions of interest by using biotinylated probes and ensure a more complete profiling of regions of interest^16^. Since the initial release of the ARTIC SARS-CoV-2 sequencing protocol early in the outbreak (Jan 22, 2020, https://artic.network/ncov-2019), amplicon-based sequencing has become the primary choice for many labs around the world and numerous commercial kits are also available^14^. This approach utilizes first-strand cDNA synthesis followed by genome amplification using viral genome specific primers to produce amplicons that are tiled across the entire genome. Sequencing adaptors and barcode indices are added, using either ligation or tagmentation-based approaches. However, the complexity of those workflows limits their scalability. Recently, Gohl et al. (2020)^17^ reported a cost-effective and highly scalable tailed amplicon method for SARS-CoV-2 sequencing, which bypasses costly and time-consuming library preparation steps.

In this study, we report an automated, high-throughput workflow for SARS-CoV-2 WGS utilizing a 2-step PCR NGS library preparation method with modified ARTIC v3 primers. Our workflow is similar to the method described by Gohl et al.^17^, where the Illumina sequencing primer-binding sites were added to ARTIC v3 gene-specific primers for use in the subsequent PCR step to add the sequencing adapters and barcode sequences. However, we made further optimizations to improve amplicon coverage balance for high-multiplexing, to minimize amplicon dropout by employing touchdown PCR^18,19^, and to integrate automated liquid handlers for high-throughput surveillance studies. Validation studies performed on clinical specimens achieved robust WGS coverage in a highly multiplexed setting with much reduced percent amplicon dropout even for the recently reported variants of concern. Using this method, we successfully converted our lower-throughput SARS-CoV-2 WGS methodology to an automated, high-throughput process. We additionally report results for over 65,000 SARS-CoV-2 whole genome sequences from clinical specimens collected in the United States from January to September 2021 as part of the ongoing US Center for Disease Control (CDC) National SARS-CoV-2 Strain Surveillance (NS3) system (https://www.cdc.gov/coronavirus/2019-ncov/variants/cdc-role-surveillance.html).

## Results

### High-throughput NGS workflow optimization

**Figure 1:**
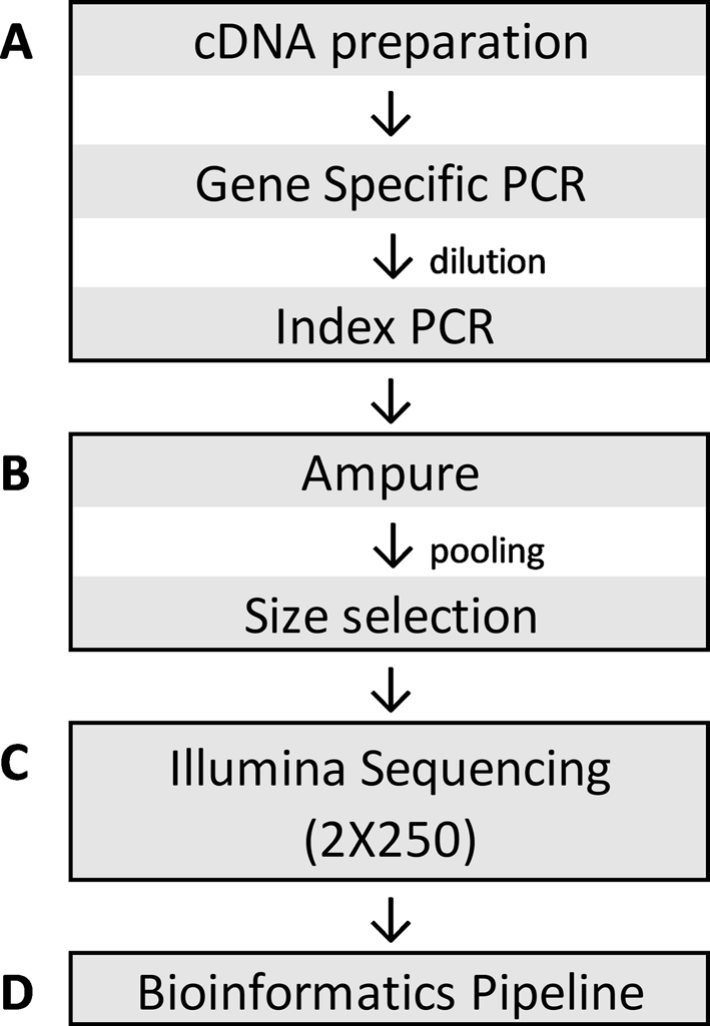
High-throughput NGS workflow. Sequencing library preparation was performed on robotic liquid handlers. (**A**) Extracted RNA of SARS-CoV-2 positive specimens were converted to cDNA and amplified using a touchdown PCR method with primers published by the ARTIC network with modifications to add Illumina sequencing primer binding sites. Specimen-specific indexed sequencing adapters were added by subsequent fusion PCR using the primer binding sites. Agilent Bravo was used for each process. (**B**) The final indexed PCR products were purified using Ampure XP beads using BlueCat BlueWasher, pooled into a single library using Hamilton Starlet, and size selected using Sage Science Blue Pippin. (**C**) The final library was sequenced on an Illumina NovaSeq 6000. (**D**) An in-house developed bioinformatics pipeline was utilized to generate consensus genomes and for variant calls relative to the reference genome MN908947.3, Wuhan-Hu-1.

**Figure 2:**
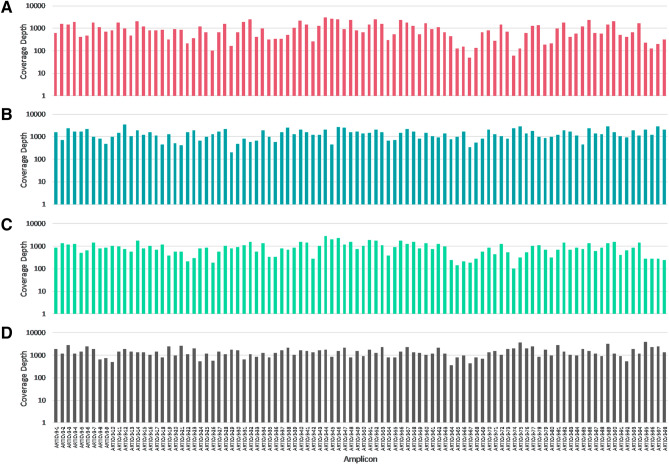
Comparison of amplicon coverage balance. A bar plot depicting the average sequencing depth of the amplicons (**A**) using equimolar primer pools, low performing amplicons were observed; (**C**) optimized modified primer pools, coverage of low performing amplicons was improved; (**B**, **D**) ARTIC v3 standard primers with even coverage. A same clinical sample set was used for plot (**A**) and (**B**) (Ct 24, n = 11); and (**C**) and (**D**) (Ct 24, n = 5). All samples were normalized to 200,000 mapped reads for comparison.

Our laboratory has developed an automated, high-throughput SARS-CoV-2 NGS workflow (Fig. 1). This workflow utilizes a 2-step PCR NGS library preparation method: (1) gene-specific PCR to amplify the SARS-CoV-2 whole genome, using primers published by the ARTIC network, with modifications to add Illumina sequencing primer binding sites; and (2) index PCR to add specimen-specific barcoded sequencing adapters by fusion PCR using the Illumina sequencing primer binding sites. We first optimized primer pools to give even coverage across the SARS-CoV-2 genome. Gene specific primers were pooled into 4 pools (pool 1A, 1B, 2A, 2B, Supplementary Table S1), and positive patient specimens with RT-qPCR cycle threshold (Ct) value of 24 (n = 11) were tested. To evaluate the coverage uniformity, we computed the average coverage of each amplicon at a normalized depth of 200,000 mapped reads. All 98 amplicons produced adequate coverage with a mean coverage of 973X (SD, 719; CV, 73.9%) (Fig. 2A). However, 7 amplicons, including 3 in the spike protein coding region, produced relatively lower coverage. When the same sample set was tested with a standard ARTIC v3 method, even coverage across the whole genome was achieved with a mean coverage of 1390X (SD, 658; CV, 47.3%) at 200,000 mapped reads (Fig. 2B).

Next, to improve coverage for these low-performing amplicons, we increased the corresponding primer concentration in the same primer pool or added the primer set to a different pool. When tested with RT-qPCR positive patient specimens (Ct 24, n = 5), the optimized primer pools improved the coverage of these low-performing amplicons by 2- to fivefold. The coverage for amplicons 26, 68, 74 and 75 was improved approximately twofold from 106X, 52X, 61X and 130X to 191X, 273X, 100X and 316X, respectively. In addition, the coverage for amplicons 29, 67 and 79 was improved approximately 4- to fivefold from 165X, 52X and 190X to 773X, 186X, and 720X, respectively. These changes resulted in improved amplicon balance with a mean coverage of 893X (SD, 514; CV, 57.6%) (Fig. 2C). For the same sample set, the standard ARTIC v3 method achieved 1470X mean coverage depth (SD, 692; CV 47.1%) (Fig. 2D).

As the SARS-CoV-2 genome has evolved, various mutations were found at the ARTIC v3 primer binding sites. Upon sequence analysis of 3506 positive samples collected and processed in January and February of 2021, we found that 96% (3367/3506) of the samples had at least one primer binding site mutation with a median of 3 (range: 1–9) mutations with the potential to impair PCR efficiency per affected sample (Supplementary Table S3).

To minimize adverse effects on sequencing coverage, we employed a modified touchdown PCR method by gradually reducing the annealing temperature from 65 °C to 55 °C (0.7 °C/s) within each PCR cycle. We compared 79 samples with two different annealing temperature settings. With this approach, the percent amplicon dropout due to primer binding site mutations was decreased from 0.50% to 0.01% (Table 1). The percent amplicon dropout was calculated from the number of amplicons that did not generate coverage divided by the total amplicon number. Each dropout amplicon was manually reviewed for the presence of a mutation at the affected primer binding site.

**Table 1:** Comparison of percent amplicon dropout by annealing temperature settings.

Clade^a^	Samples tested (N)	% Amplicon dropout:	% Amplicon dropout:
		Annealing at 65 °C	Annealing at 65–55 °C^b^
20A	12	0.34	0
20B	10	0.51	0
20C	3	1.7	0.34
20G	24	0.21	0
20H (Beta, V2)	1	2.04	0
20I (Alpha, V1)	9	0	0
21C (Epsilon)	17	1.08	0
21F (Iota)	3	0	0
Total	79	0.5	0.01

^a^SARS-CoV-2 clades were assigned with Nextclade v1.3.0 (https://clades.nextstrain.org/).

^b^Touchdown PCR gradually reduced the annealing temperature from 65 °C to 55 °C (0.7 °C/second) within each cycle.

### Assay precision

**Figure 3:**
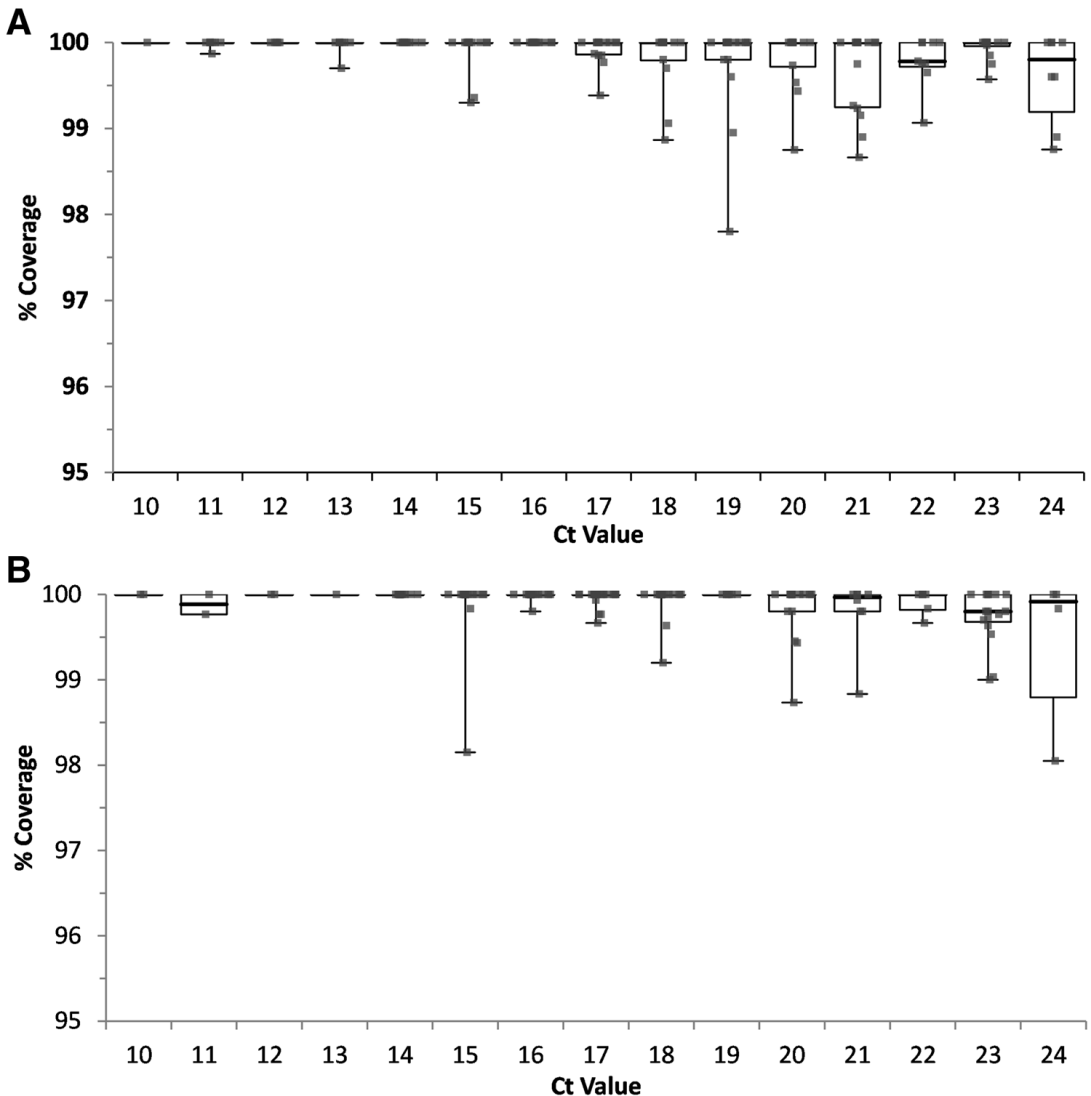
Percent genome coverage for intra-assay (**A**) and inter-assay (**B**) precision samples. Square dots indicate the average percent coverage of the SARS-CoV-2 genome for each replicate. The box plot shows the medians, interquartile ranges (IQR) and 1.5 × IQR at each Ct value. Ct values were rounded down.

We assessed intra-assay precision using 188 unique specimens with Ct values between 10 and 25 run in 3 replicates. Most (523/564; 92.8%) sequences met quality control metrics (Fig. 3A); 175/188 (93.1%) unique samples met quality control requirements for 2 or more replicates and were utilized for lineage and clade comparison. All 175 samples had concordant clade and lineage assignments for all replicates (Table 2). Additionally, spike protein amino acid substitutions were analyzed for all 175 samples. On average, 7.3 ± 2.9 substitutions were detected per sample. There was 99.8% qualitative (1272/1275, 95% CI: 99.3–99.9%) agreement when the minority variant frequency was > 20% (Supplementary Fig. S1 A). The average %CV for variant frequency between replicates was 0.5% (min CV: 0.0%; max CV: 6.7%).

**Table 2:** Intra- and inter-assay concordance of clade and lineage assignment using the automated, high-throughput SARS-CoV-2 NGS workflow.

Clade/lineage^a^	Number of samples		
	Intra-assay precision	Inter-assay precision	Total tested/Total correct
20A/B.1	5	1	6/6
20A/B.1.189	0	1	1/1
20A/B.1.232	0	1	1/1
20A/B.1.234	0	3	3/3
20A/B.1.243	0	3	3/3
20A/B.1.525	2	1	3/3
20A/B.1.539	0	1	1/1
20A/B.1.628	1	0	1/1
20B/B.1.1	0	1	1/1
20B/B.1.1.222	1	0	1/1
20B/B.1.1.231	1	0	1/1
20B/B.1.1.265	1	0	1/1
20B/B.1.1.316	1	0	1/1
20B/B.1.1.318	2	0	2/2
20B/B.1.1.345	0	1	1/1
20B/B.1.1.348	1	1	2/2
20B/B.1.1.434	2	0	2/2
20B/B.1.1.519	8	15	23/23
20B/P.2	0	1	1/1
20B/R.1	0	4	4/4
20C/B.1	3	0	3/3
20C/B.1.1	3	0	3/3
20C/B.1.324	1	0	1/1
20C/B.1.427	2	5	7/7
20C/B.1.429	2	16	18/18
20C/B.1.517	6	1	7/7
20C/B.1.526	8	4	12/12
20C/B.1.526.1	3	4	7/7
20C/B.1.526.2	9	5	14/14
20C/B.1.575	2	2	4/4
20C/B.1.637	1	0	1/1
20G/B.1.2	12	38	50/50
20G/B.1.596	4	6	10/10
20I (Alpha, V1)/B.1.1.7	77	44	121/121
20I (Alpha, V1)/Q.4	2	0	2/2
20I (Alpha, V1)/Q.8	1	0	1/1
20J (Gamma, V3)/P.1	5	1	6/6
21D (Eta)/B.1.525	2	0	2/2
21F (Iota)/B.1.526	5	0	5/5
21H/B.1.621	2	0	2/2
Total	175	160	335/335

^a^SARS-CoV-2 clades were assigned with Nextclade v1.3.0 (https://clades.nextstrain.org/) and lineages were assigned with Pangolin v3.1.11 (https://pangolin.cog-uk.io/).

We then assessed inter-assay precision for 168 unique specimens with Ct values between 10 and 25 over 3 independent runs. Overall, 479/504 (95.0%) sequences met quality control requirements (Fig. 3B). Over 95% (160/168) of samples had valid sequence data for 2 or more replicates and were utilized for clade and lineage comparisons. All 160 samples had concordant clade and lineage assignments (Table 2). On average, there were 5.9 ± 3.2 spike protein amino acid substitutions per sample with 99.5% (946/951, 95% CI: 98.8–99.8%) qualitative agreement between runs when the minority variant frequency was present in > 20% of the reads (Supplementary Fig. S1 B). The average %CV for the variant frequency between replicates was 0.9% (min CV: 0.0%; max CV: 20.3%).

### Assay sensitivity

**Figure 4:**
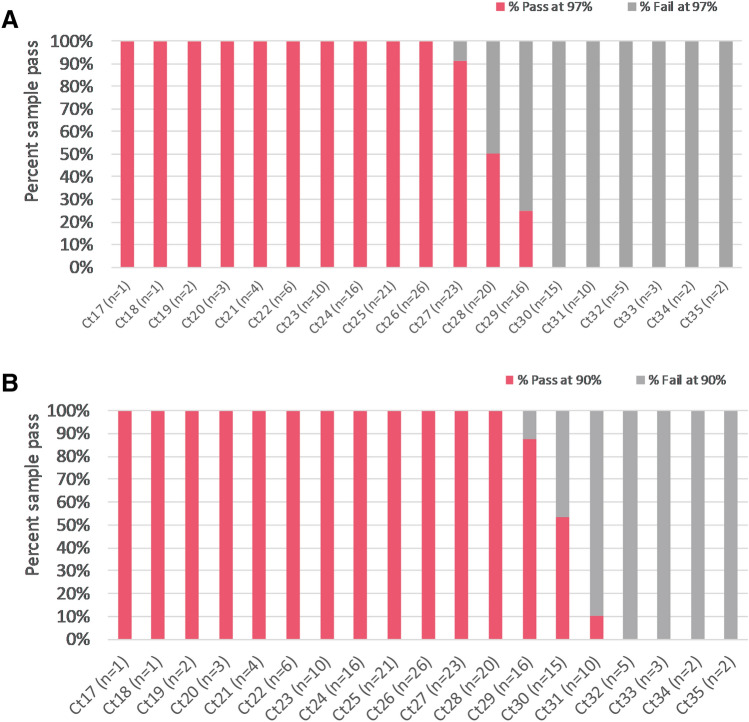
Sequencing pass rate (%) for Ct values between 17 and 35. Percent sample pass at over 97% consensus sequence coverage (**A**), and at over 90% consensus sequence coverage (**B**). Sample numbers tested per Ct are indicated in parentheses. Ct values were rounded down.

To demonstrate that our high-throughput workflow offers adequate sensitivity to yield complete SARS-CoV-2 genomes, we determined the limit of detection. The limit of detection was defined as the highest Ct value that yielded valid sequence data with ≥ 97% SARS-CoV-2 genome coverage for at least 95% of the specimens tested. A total of 39 unique samples with Ct values between 17 and 32 were serially diluted, yielding 186 samples with Ct values between 17 and 35. The percent of samples that yielded ≥ 97% consensus sequence ranged between 91 and 100% up to a Ct value of 27 (Fig. 4A). Only 50% (10/20) and 25% (4/16) of samples with Ct values of 28 and 29, respectively, passed. Of note, 100% (20/20) of samples with Ct values of 28 and 88% (14/16) of those with Ct values of 29, generated > 90% consensus sequence. Thus, 98.7% (147/149) of samples with Ct values less than 30 yielded > 90% consensus sequence (Fig. 4B). When over 90% consensus sequence was generated, there was 100% concordance for clade and lineage assignments.

### Accuracy study

Next, we assessed the accuracy of the consensus sequences generated by this workflow. Three commercial synthetic RNA positive controls (clade Alpha, Beta, and Gamma variants; Twist Bioscience) were tested 12 times each, using 4 replicates per set-up in 3 independent set-ups, by 3 different scientists. Of note, the synthetic RNA controls do not cover 100% of the SARS-CoV-2 genome, and amplicon dropout was expected. In 36 trials, the mean percent consensus sequence was 95.1% (min 93%; max 98%). All controls resulted in 100% positive percent agreement for clade and lineage assignments (95% CI: 90.4%, 100.0%) and 100% for spike protein mutations that were detected when the minor allele frequency was over 20% (95% CI: 98.8%, 100.0%).

In addition, a total of 84 qRT-PCR negative samples were evaluated, in 3 independent set-ups using 28 negative samples per run, by 3 different scientists. All negative samples gave negative results, with mean amplicon coverage of 0.2 (min 0.0%, max 0.6%) relative to the PCR plate average, yielding 100% sample-level negative percent agreement (95% CI: 95.6%, 100.0%).

### Robustness study

To assess assay robustness, a total of 2688 samples, including 1662 unique SARS-CoV-2 positive clinical specimens, 21 synthetic positive controls, 49 SARS-CoV-2 negative specimens and 56 no-template controls (NTCs), were sequenced in a single Illumina NovaSeq sequencing run. Over 94.3% (2416/2562) of the total number of sequenced samples and 95.3% (1584/1662) of the unique clinical specimens passed quality control with a median coverage of 2478X. All negative specimens gave a negative result (median coverage 2.4X, < 0.1% relative coverage); and all positive controls gave the expected lineage and clade assignments. To monitor possible sample contamination, 2 NTCs per 96 samples were included. Although 54 of the 56 NTCs gave a negligible number of reads (0.025% relative coverage to the plate mean), 2 NTCs showed 5.7% and 8.2% relative coverage indicating possible sample crossover. To assess the effects of the NTC contamination on assay accuracy, we compared the sequences to those obtained from the same clinical specimens (n = 1446) previously sequenced using the standard ARTIC v3 workflow on Illumina MiSeq sequencers. Concordance was 99.9% (1445/1446) for clade, and 99.8% (1444/1446) for lineage assignment (Supplementary Tables S4, S5). Moreover, there was 100% clade and lineage concordance for all positive clinical specimens processed in the same 384-well plates with the 2 higher-coverage NTC wells. These results indicate that a low degree of NTC contamination is unlikely to interfere with accurate consensus sequence generation or with clade and lineage assignments.

Next, we analyzed the proportion of samples that generated 100% consensus coverage out of the samples that passed coverage QC (Table 3). With the optimized high-throughput workflow, over 95.9% of samples generated a complete consensus (range: 83.0% to 100% per clade). When the same set of samples were analyzed with a standard ARTIC v3 workflow, the proportion of samples with complete consensus sequence was much lower, 66.1% (range 0% to 100.0% per clade). In some variants sequenced with the ARTIC v3 workflow (clades Beta, Delta, Epsilon, and Lambda), amplicon dropouts were observed in almost all cases; dropout was resolved by employing our high-throughput workflow utilizing touchdown PCR.

**Table 3:** Proportion of SARS-CoV-2 samples generating complete consensus sequence with the automated, high-throughput SARS-CoV-2 NGS workflow, by clade.

Clade^b^	Samples generating complete consensus sequence with each workflow, % (complete/total)^a^	
	High-throughput workflow^c^	ARTIC v3 workflow^d^
19B	100 (3/3)	0 (0/3)
20A	83.0 (44/53)	62.2 (33/53)
20B	96.2 (102/106)	59.4 (63/106)
20C	88.7 (87/98)	47.9 (47/98)
20G	96 (168/175)	81.1 (142/175)
20H (Beta, V2)	100 (7/7)	0 (0/7)
20I (Alpha, V1)	96.9 (691/713)	78.2 (558/713)
20 J (Gamma, V3)	100 (44/44)	45.4 (20/44)
21A (Delta)	100 (1/1)	0 (0/1)
21C (Epsilon)	98.0 (99/101)	0.99 (1/101)
21D (Eta)	100 (10/10)	100 (10/10)
21F (Iota)	97.7 (128/131)	61.8 (81/131)
21G (Lambda)	100 (1/1)	0 (0/1)
21H	100 (3/3)	66.6 (2/3)
Total	96.0 (1388/1446)	66.1 (957/1446)

^a^Complete consensus sequence as defined by obtaining ≥ 97% SARS-CoV-2 genome coverage.

^b^SARS-CoV-2 clades were assigned with Nextclade v1.3.0 (https://clades.nextstrain.org/).

^c^Samples were sequenced on the Illumina NovaSeq 6000 with the SP reagent kit using 2 × 251 cycles.

^d^Samples were sequenced on the Illumina MiSeq with the 600 cycle v3 kit using 2 × 251 cycles.

### Large-scale surveillance study

From January to September 2021, over 65,000 SARS-CoV-2 qRT-PCR-positive or transcription-mediated amplification (TMA)-positive specimens were successfully sequenced in our laboratory on behalf of the CDC National SARS-CoV-2 Strain Surveillance (NS3) project (https://www.aphl.org/programs/preparedness/Crisis-Management/COVID-19-Response/Pages/Sequence-Based-Surveillance-Submission.aspx). Specimens were collected from all 50 US states, the District of Columbia and Puerto Rico. The leading 10 states that cumulatively contributed two-thirds (66%) of the specimens over this study period were Texas (15.1%), California (11.8%), Florida (10.5%), Pennsylvania (6.1%), Nevada (5.8%), Massachusetts (4.4%), Illinois (4.1%), Connecticut (4.1%) and Ohio (4.0%). Between January and the end of May 2021, the sequencing libraries were constructed using the modified ARTIC v3 protocol as described in “Methods”. Subsequently, libraries were constructed using the high-throughput workflow designed to improve scalability, as reported in this study. Cumulatively, the Delta variant of concern (VOC) was detected at the highest frequency (35%), because of the steep increase in prevalence of this variant after June 2021 (https://covid.cdc.gov/covid-data-tracker/#variant-proportions). Viruses in clade Alpha were the second-most prevalent, accounting for 29% of specimens tested, reflecting their high prevalence before June 2021. Other variants being monitored (VBMs) were detected at much lower frequencies, including Epsilon (5%), Iota (5%), and Gamma (4%) (Supplementary Table S8). Beta, Lambda, Eta, and Kappa variants were found in less than 1% of the specimens sequenced.

**Figure 5:**
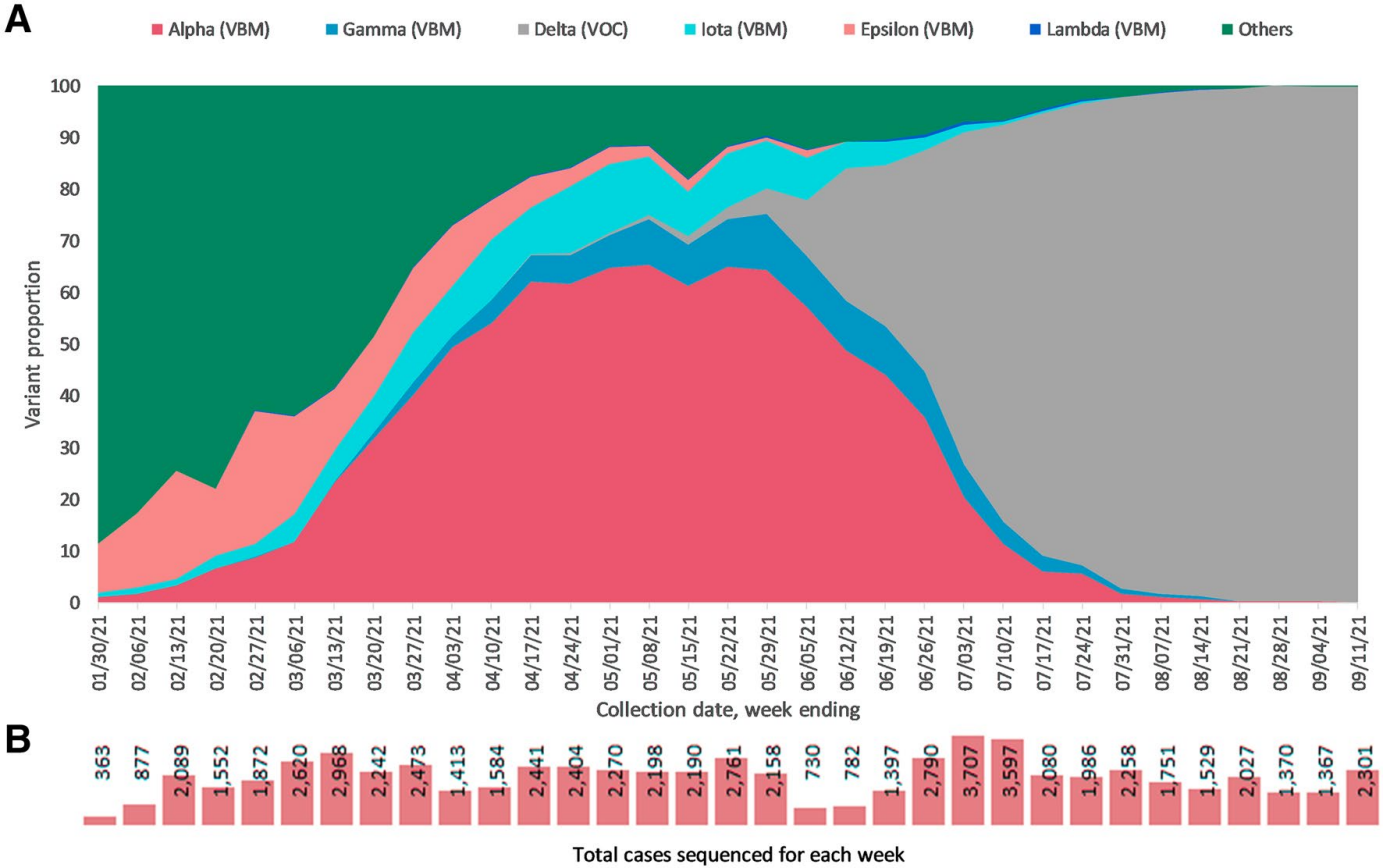
Weekly proportions of SARS-CoV-2 variants. (**A**) Variant proportions were calculated against total cases sequenced each week. (**B**) Total number of cases sequenced for each week is indicated with scale bar.

We tracked the proportions of VBM and VOC variants on a weekly basis (Fig. 5). The Epsilon variant was predominant (25.6%) in January and February 2021, then subsequently declined in frequency as the Alpha and Iota variants became more prevalent (65.3% and 13.3%, respectively) through May 2021. The Delta variant subsequently emerged in May 2021, rapidly increasing in prevalence to reach 99.8% of all specimens sequenced by mid-September 2021. These trends were consistent with national variant trends as reported by the CDC (https://covid.cdc.gov/covid-data-tracker/#variant-proportions).

## Discussion

Pathogen WGS has been employed to investigate cases of foodborne disease outbreaks, methicillin-resistant *Staphylococcus aureus* (MRSA) outbreaks, and outbreaks of other bacterial and fungal pathogens^20^. However, these studies were retrospective in nature. Rapid high-throughput viral genomic sequencing is being increasingly used for outbreak research and is changing the practice of epidemiology^21^. For example, advances in high-throughput sequencing facilitated near-real time WGS to investigate the 2014–2015 Ebola virus outbreak in West Africa^22^. In the present COVID-19 pandemic, rapid SARS-CoV-2 WGS, combined with epidemiologic methods, identified or excluded nosocomial transmission events to directly affect outbreak management in real time^23^. Hospital-based WGS set up in collaboration with public health departments has also facilitated real-time outbreak investigations and identification of transmission chains^24^. At a national level, the Coronavirus Disease 2019 (COVID-19) Genomics UK Consortium (COG-UK) has developed high-throughput sequencing and analysis workflows to generate hundreds of thousands of SARS-CoV-2 genomic sequences, enabling large-scale genomic epidemiology to inform public health response (https://www.cogconsortium.uk/). In the United States, the CDC established the SARS-CoV-2 Sequencing for Public Health Emergency Response, Epidemiology and Surveillance (SPHERES) initiative in the early stages of the pandemic to accelerate the use of real-time pathogen sequence data and molecular epidemiology for the COVID-19 pandemic response (https://www.cdc.gov/coronavirus/2019-ncov/variants/spheres.html).

Large scale and high-throughput WGS of SARS-CoV-2 is essential to enable real-time epidemiologic surveillance and to better understand the evolution and spread of the pandemic, improve epidemiologic tracking, and enhance treatment and prevention strategies for COVID-19. To date, several groups have developed different protocols and workflows for SARS-CoV-2 WGS by NGS. However, most of the published studies are small in study sample size, low in throughput or may require complex enzymatic steps^11,12,25–29^. In one study, the commercially available COVIDSeq test (Illumina) was scaled up to sequence 752 clinical samples in duplicate for a total of 1536 samples and controls on a NovaSeq 6000 instrument using an S4 flow cell^30^. However, this study described only a single sequencing run and did not utilize an automated high throughput workflow. In the current study, we were able to use the lower output and less expensive SP flow cell to sequence over 2600 samples.

Here, we report an automated, high-throughput workflow for SARS-CoV-2 WGS optimized for large scale surveillance studies, utilizing a 2-step PCR NGS library preparation method with modified ARTIC protocol v3 primers. Validation studies performed on 1711 unique clinical samples demonstrated high precision (100% inter- and intra-assay precision) and accuracy (100% PPA and 100% NPA). Slightly reduced, but adequate sensitivity was achieved in comparison to the lower-throughput in-house validated ARTIC v3 protocol: near complete consensus sequence was generated for samples of qRT-PCR Ct values less than 28 using the 2-step PCR workflow (Fig. 4) and with samples of Ct less than 30 by the ARTIC v3 workflow (data not shown).

For large-scale surveillance studies, integration of robotic liquid handlers is a crucial component. Various automation platforms are available for NGS library preparation. Upon evaluation of frequently utilized liquid handlers, the Agilent Bravo platform produced the most consistent results for our workflow in a 384-well format and was used for library preparation. As noted by Gohl et al.^17^, a 2-step PCR method increased primer dimer formation which can result in lower sequencing quality. To remove any primer dimer present, we implemented 3 library cleaning steps: (1) Ampure bead clean up using a centrifugal washer in 384-well format; (2) manual Ampure bead clean up of a pooled library; (3) size selection using 1.5% gel on a BluePippin station prior to loading on the Illumina NovaSeq 6000. With these stringent clean-up steps, our validation runs achieved over 90% (range 90.6%-93.4%) high base call Phred quality scores (Q30 or higher).

The ARTIC amplicon-based whole-genome amplification of SARS-CoV-2 is considered a highly sensitive and accurate method and is currently employed by many laboratories around the world. One problem associated with amplification of the SARS-CoV-2 genome is amplicon dropout which can be caused when a given primer cannot stably hybridize to its specific complementary sequence binding site because of novel mutations at the primer binding site. As the SARS-CoV-2 genome has evolved, various mutations were found in ARTIC v3 primer binding sequence^31^. This type of dropout often requires redesign and revalidation of primers. To minimize amplicon dropout, we employed a modified touchdown PCR method which reduced amplicon dropout (Table 1). The touchdown PCR method is expected to increase PCR specificity by reducing the amplification of spurious amplicons at higher annealing temperatures, while at the same time maintain a competitive advantage for the targeted amplicons and increase their yield, even in the presence of potential primer-binding site mutations, as the annealing temperature is gradually reduced^19^. The effects of this improvement were most prominent for Beta, Delta, Epsilon, and Lambda clade specimens, achieving 100% consensus sequence coverage in most trials (Table 3). On the other hand, for these 4 clades, when analyzed by the standard ARTIC v3 workflow, nearly all samples failed to achieve full consensus sequence coverage due to primer binding site mutations causing amplicon dropout (Table 3, Supplementary Table S6). We extended our analysis to samples collected between May and September of 2021 to show that the benefit of using the touchdown PCR method is not limited to the validation sample set. Similar results were observed for Beta, Delta, Epsilon, and Lambda clade specimens with 95% of samples generating a complete consensus sequence using the high-throughput workflow whereas only 2.9% of samples generated complete consensus sequence by the ARTIC v3 workflow (Supplementary Table S7). The ARTIC network has published a newer ARTIC v4 primer set, which was designed to avoid current high-frequency mutations, with the goals of minimizing amplicon dropout and producing high-accuracy variant calling. However, analytical validation is required prior to using this new primer set in surveillance studies. We envision that a touchdown PCR method can be easily adapted to any workflow and can avoid frequent primer redesign and validation.

The 2-step PCR method generated 30% lower coverage compared to the ARTIC v3 method when the per sample coverage was averaged across all amplicons and normalized to 200,000 mapped reads per sample (Fig. 2A,B). We postulate that this difference may be due to the higher primer dimer formation during the 2-step PCR as mentioned by Gohl et al.^17^. Although the normalized coverage was reduced by approximately 30%, on average, for the 2-step PCR method, the normalized coverage still exceeded the minimum coverage necessary to reliably generate a viral genome consensus sequence or to detect viral variants present in at least 50% of the reads (consensus calling threshold). Indeed, the absolute coverage obtained even when processing > 2600 samples on the NovaSeq sequencer (median coverage 2478X) greatly exceeded the absolute coverage obtained for ARTIC v3 runs on the MiSeq sequencer (median coverage 1098X with a batch size of 192). Therefore, these differences in normalized coverage have minimal practical impact on the ability of the 2-step PCR method to generate reliable whole genome consensus sequences or to detect variants.

In summary, building on SARS-CoV-2 WGS methodological improvements reported elsewhere^17^, we report on the development and the validation of an automated 2-step PCR, high-throughput NGS workflow for SARS-CoV-2 WGS with high sensitivity and accuracy with much less amplicon dropout, which we have implemented for high-throughput SARS-CoV-2 genomic surveillance. The combination of automation and optimized bench and analysis workflows has enabled efficient, large-scale SARS-CoV-2 surveillance studies.

## Methods

### Sample collection

We obtained remnant RNA stored at − 80 °C, extracted from deidentified clinical specimens previously tested for SARS-CoV-2 by qRT-PCR at Quest Diagnostics between February and August 2021. For workflow development, 16 positive samples with a Cycle threshold (Ct) value 24 were processed for sequencing. Analytical method validation studies were performed on a random set of 1711 unique clinical specimens collected in March and April of 2021. In addition, 3 twist Synthetic SARS-CoV-2 RNA controls (Twist Bioscience, San Francisco, CA) and 24 negative samples (Ct > 50 by qRT-PCR) were included for accuracy studies.

For surveillance studies, a random set of residual nasopharyngeal or oropharyngeal swab specimens from SARS-CoV-2 qRT-PCR positive or transcription mediated amplification (TMA) positive specimens, performed at Quest Diagnostics, were collected across the United States between January and September 2021. For SARS-CoV-2 qRT-PCR positive sample selection, Ct < 25 was applied for qualification. In accordance with ethical requirements and in accordance with US Department of Health and Human Services guidelines for the use of deidentified specimen remnants in research studies, specimens were deidentified prior to the study and were limited to remnant extracted RNA from discarded specimens previously submitted for commercial testing.

### Two-step PCR library preparation

For cDNA synthesis a mixture of 10.8 μl extracted RNA and 2.7 μl SuperScript IV VILO master mix (Thermo Fisher Scientific, Waltham, MA) was incubated at 25 °C for 10 min followed by 50 °C for 10 min and 80 °C for 5 min. To amplify the entire SARS-CoV-2 genome, four gene specific PCR reactions were performed using each of the four primer pools (Supplementary Table S1). The primers were based on the ARTIC v3 primer set^32^ with modifications to add Illumina sequencing primer binding sites: 5’-ACACTCTTTCCCTACACGACGCTCTTCCGATCT-3’ was added to 5’ of all forward primers; 5’-GTGACTGGAGTTCAGACGTGTGCTCTTCCGATCT-3’ was added to 5’ of all reverse primers. To avoid primer interactions, 11 of the ARTIC v3 primers were replaced with primers reported by Itokawa et al.^33^: forward primers for amplicons 21, 36, and 89; reverse primers for amplicons 1, 9, 13, 15, 32, 38, 76, and 89. For the gene specific PCR reactions, 1.5 μl of cDNA was used in 15 μl reaction mixture of Q5 Hot START DNA Polymerase kit (New England Biolabs, Ipswich, MA): 3 μl of 5X buffer, 0.3 μl of 10 mM dNTPs, 0.15 μl of polymerase, 2.5 μl of 10 μM primer pool (1A, 1B, 2A, or 2B), and 7.55 μl nuclease-free water. The initial denaturation occurred at 98 °C for 30 s followed by 30 cycles of 98 °C for 15 s, 65 °C for 40 s, 55 °C for 40 s (ramp rate of 0.7 °C/sec), 72 °C for 2.5 min and the final extension at 72 °C for 2 min. The four PCR products for the same clinical samples were combined and diluted 1:100 in nuclease-free water. The diluted product was amplified with index primers that add sample index as well as the Illumina sequencing adapters needed for sequencing on the Illumina sequencers. The forward and reverse index primers had the following structure:

forward, 5’-AATGATACGGCGACCACCGAGATCTACAC-[12nt-i5-index]-ACACTCTTTCCCTACACGACGCTC-3’;

reverse, 5’-CAAGCAGAAGACGGCATACGAGAT-[12nt-i7-index]-GTGACTGGAGTTCAGACGTGTGC-3’.

For the index PCR, 3 μl of the diluted gene specific PCR product was used in 15 μl reaction mixture of KAPA HiFi HotStart ReadyMix (Kapa Biosystems, Wilmington, MA): 7.5 μl of 2X buffer, 2 μl of 5 μM dual-indexed primer mix, and 2.5 μl nuclease-free water, using the following program: 98 °C for 45 s followed by 15 cycles of 98 °C for 15 s and 60 °C for 30 s, 72 °C for 30 s, and the final extension at 72 °C for 1 min.

The indexed products were purified with 0.7X Ampure XP beads (Beckman Coulter, Beverly, MA) on a BlueWasher 384-well format (BlueCatBio, Concord, MA) and quantified using Qubit dsDNA Broad-Range reagent (Thermo Fisher Scientific, Waltham, MA) on a Tecan Infinite F200 Pro, 384 well reader (Tecan, Männedorf, Switzerland). The purified products were pooled, 250 ng per sample, into a single library using Hamilton STARlet (Hamilton, Reno, NV) and was concentrated with 0.6X Ampure XP beads and size selected using a 1.5% gel cassette on the BluePippin (Sage Science, Beverly, MA) according to the manufacturer protocol. The size selected library was used for loading on a MiSeq with 192 samples per run or on a NovaSeq 6000 with 1536 or 2688 samples per run.

### ARTIC v3 library preparation

The cDNA synthesis and PCR steps were identical to the original ARTIC v3 protocol (https://www.protocols.io/view/ncov-2019-sequencing-protocol-bbmuik6w) with minor modifications. In brief, RNA samples were reverse transcribed using the SuperScript IV kit (ThermoFisher, Waltham, MA) and random hexamers. RNA samples with Ct < 18 were diluted 50-fold (Ct 12–15) or tenfold (Ct 15–18) before use. In brief, 11 μl RNA samples were mixed with 1 μl 50 μM random hexamers and 1 μl 10 mM dNTPs. The mixture was incubated for 5 min at 65 °C and placed directly on ice. After 2–3 min, 7 μl enzyme mix containing 4 μl 5X Buffer, 1 μl 0.1 M DTT, 1 μl RNaseOUT RNase inhibitor, and 1 μl SuperScript IV reverse transcriptase was added to the samples. The reactions were incubated at 42 °C for 50 min, and at 70 °C for 10 min followed by cooling to 4 °C. For the multiplex PCR reactions, 2.5 μl of the cDNA was used in 25 μl reaction mixture of Q5 Hot START DNA Polymerase kit (New England Biolabs, Ipswich, MA): 5 μl 5X buffer, 0.5 μl 10 mM dNTPs, 0.25 μl polymerase and 3.6 μl 10 μM primer pool 1 or pool 2, and 13.15 μl nuclease-free water. The primer pools were based on the ARTIC v3 primer pool scheme with further optimization to enhance low-performing amplicons (Supplementary Table S2). The thermal program was identical to the original ARTIC protocol: 30 s polymerase activation at 98 °C followed by 30 cycles of 15 s denaturing at 98 °C and 5 min annealing and extension at 65 °C. The PCR products in pool 1 and 2 reactions for same clinical samples were combined and purified with 1X AMPure XP and quantified with Qubit Broad Range Kit on SpectraMax (Molecular Devices, San Jose, CA).

The purified PCR products were converted to Illumina sequencer-compatible libraries using the Twist Library Preparation kit (Twist Bioscience, San Francisco, CA). In brief, 300 ng of purified PCR products in 15 μl were end-repaired in 25 μl of end repair reaction mixture: 2.5 μl 10X buffer, 5 μl enzyme mix, and 2.5 μl water and incubated at 20 °C for 30 min, and at 65 °C for 30 min. Illumina adapter was ligated to the end-repaired mix by adding 25 μl ligation master mix: 10 μl 5X buffer, 5 μl enzyme mix, 2.5 μl 5 μM adapter, and 7.5 μl water and incubated at 20 °C for 15 min. The ligated products were purified using 0.8X Ampure XP beads for subsequent index PCR using KAPA HiFi HotStart ReadyMix in 50 μl reaction volume with 6 μl 5 μM index primers using the following program: 98 °C for 45 s followed by 8 cycles of 98 °C for 15 s, 60 °C for 30 s, and 72 °C for 30 s, and the final extension at 72 °C for 1 min. The indexed products were purified using 1X Ampure XP beads, quantified using Qubit Broad range reagent, and normalized by pooling 400 ng per sample. The pooled library was used for MiSeq loading with 192 samples per run.

### Sequencing

For sequencing on a NovaSeq 6000 (Illumina, San Diego, CA), the final library was diluted to 1700 pM and PhiX was spiked at 18%. The combined library was denatured with 0.2 N NaOH and neutralized with 0.4 N Tris–HCl and sequenced on a NovaSeq 6000 SP Reagent kit using 2 × 251 cycles. For sequencing on a MiSeq (Illumina, San Diego, CA), the final library was diluted to 2 nM, and spiked in 20% PhiX, denatured with 0.2 N NaOH and neutralized with 0.2 N HCl, and diluted with Illumina HT1 buffer to 10 pM and sequenced using a MiSeq 600 cycle v3 kit, 2 × 251 cycles.

### Analysis

For MiSeq runs, the paired-end fastq files generated by MiSeq Reporter 2.5.1.3 were used. For NovaSeq 6000 runs, bcl files generated by Illumina NovaSeq RTA v.3.4.4 were converted to fastq by using Illumina bcl2fastq v2.20.0.422 software without lane splitting. For a batch size of 1536, the resulting fastq files were down-sampled by 50%. The fastq files were mapped to SARS-CoV-2 (MN908947.3) supplemented with the human genome (GRCh37) reference sequence using BWA v0.7.17-r1188 (Supplementary Fig. S2)^34^. PCR primer sequences were trimmed from the mapped reads to MN908947.3 using iVar v1.3.1 or version 1.2.2 prior to February 2021 (Supplementary Fig. S2)^35^. iVar was also used for variant calling and consensus sequence creation with minimum sequencing depth set to 10 reads for MiSeq data, and a minimum variant frequency of 0.5. For NovaSeq data, coverage equivalent to the number of mapped reads divided by 20,000 was required for consensus sequence generation and variant calling. Reads sorting and filtering were performed by SAMtools v1.9 (Supplementary Fig. S2)^36^. Coverage depth of each nucleotide position was determined with BEDTools v2.29.2 (Supplementary Fig. S2)^37^. Percent genome coverage was determined by counting the number of nucleotides meeting the minimum coverage requirement divided by the total SARS-CoV-2 genome length excluding the 5’ and 3’ ends not covered by the amplicon panel. For clade and lineage assignment, Nextclade version 1.3.0 and Pangolin version 3.1.11 with pangoLEARN 2021–08-24 were used. Clade and lineage assignments initially performed with earlier versions of Nextclade and Pangolin were subsequently reprocessed with the above-indicated versions.

### Analytical method validation

Using the optimized workflow, we performed analytical method validation studies on 1711 unique clinical specimens collected in March and April of 2021, along with positive and negative controls. A sequencing batch size of 1536 or 2688 was employed using 384-well plates (4 plates for the 1536 batch size and 7 plates for the 2688 batch size). When the batch size was 1536, the resulting fastq files were down-sampled by 50% before analysis. For sample quality control, we set a minimum sequencing coverage threshold of 10X spanning at least 97% SARS-CoV-2 genome.

## Supplementary Information

Supplementary Information 1.

Supplementary Information 2.

## Data Availability

NovaSeq runs and sequence IDs are listed in Supplementary Materials file “Sequenced_sample_list.xlsx”. Fasta consensus sequences in compressed (zip) file format will be provided upon request. Large scale surveillance sequencing was performed for the CDC National SARS-CoV-2 Strain Surveillance (NS3) project (https://www.aphl.org/programs/preparedness/Crisis-Management/COVID-19-Response/Pages/Sequence-Based-Surveillance-Submission.aspx). Consensus sequences were submitted to the CDC and were subsequently deposited by the CDC in the GISAID database (https://www.gisaid.org/); originating lab: Quest Diagnostics Incorporated. Sequences submitted by Quest Diagnostics as part of this project may be retrieved from the GISAID search interface by using a virus name search, keyword “QDX”.
