# Supplementary material for: Development and validation of a high throughput SARS-CoV-2 whole genome sequencing workflow in a clinical laboratory
Source: Sci Rep. 2022 Feb 8;12:2054. doi: 10.1038/s41598-022-06091-0 (PMC8826425; doi:10.1038/s41598-022-06091-0)
Supplement: Supplementary file 1 — Supplementary Information 1. [file 41598_2022_6091_MOESM1_ESM.pdf]

Development and validation of a high throughput SARS-CoV-2 whole genome sequencing workflow in a clinical laboratory

Sun Hee Rosenthal,[1,†] Anna Gerasimova, [1,†] Rolando Ruiz-Vega, [1,†] Kayla Livingston, [1,†] Ron M. Kagan, [1,*] Yan Liu[1], Ben Anderson[1], Renius Owen[1], Laurence Bernstein[1], Alla Smolgovsky[1], Dong Xu[1], Rebecca Chen[1], Andrew Grupe[1], Pranoot Tanpaiboon[1], Felicitas Lacbawan[1,*]

[1]Quest Diagnostics, San Juan Capistrano, CA 92675 USA

†These authors have contributed equally to this work.

*Corresponding authors

Ron.M.Kagan@questdiagnostics.com

Felicitas.L.Lacbawan@questdiagnostics.com

SUPPLEMENTARY MATERIALS

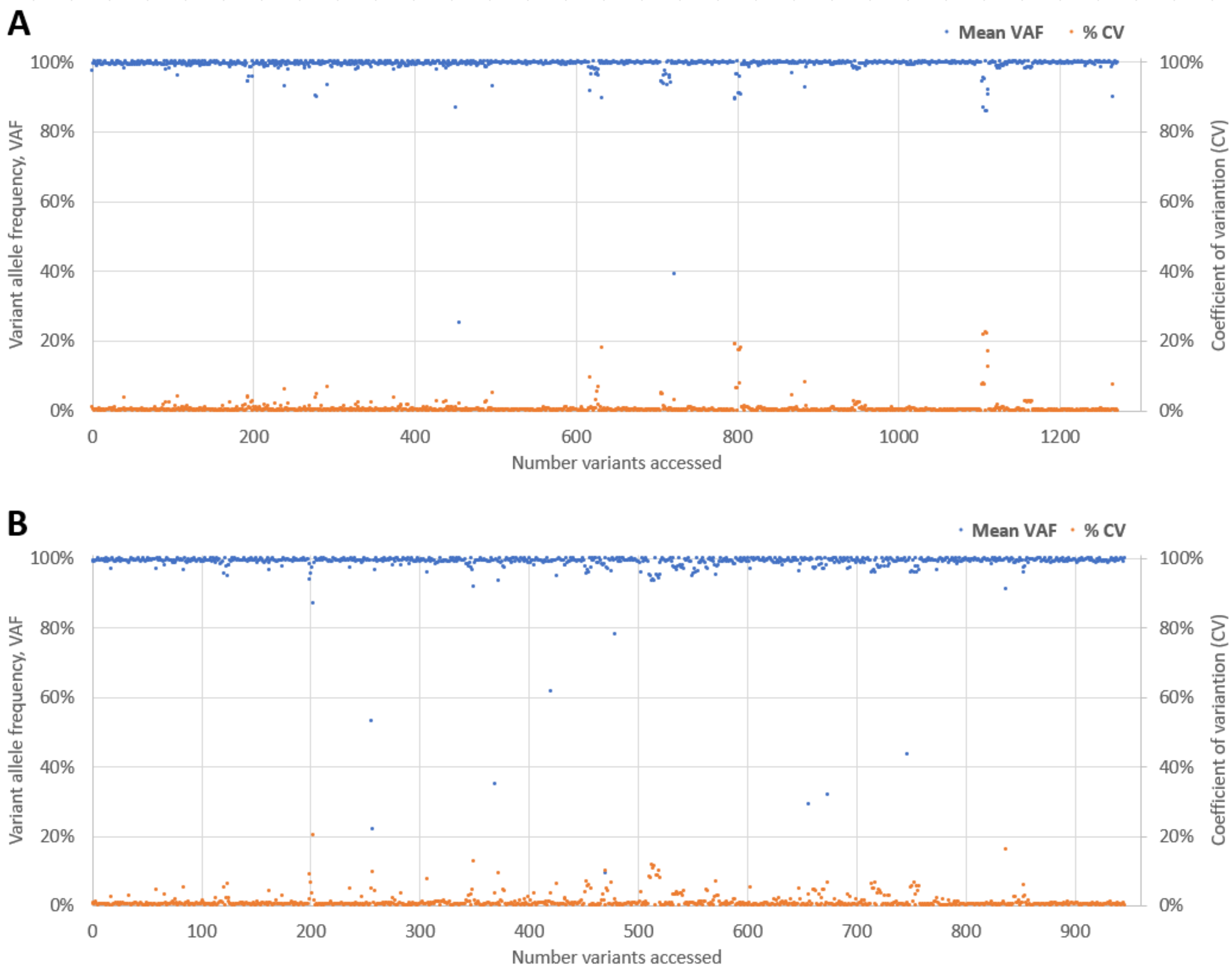

Figure S1. **Precision studies using the automated, high-throughput SARS-CoV-2 NGS workflow.** (A) Intra-assay precision of 1,272 variants from 175 specimens repeated 3 times. The mean of the detected variant frequencies (blue dot) and coefficients of variations (orange dot) are shown. (B) Inter-assay precision of 946 variants from 160 specimens replicated 3 times. The mean of the detected variant frequencies (blue dot) and coefficients of variations (orange dot) are shown.

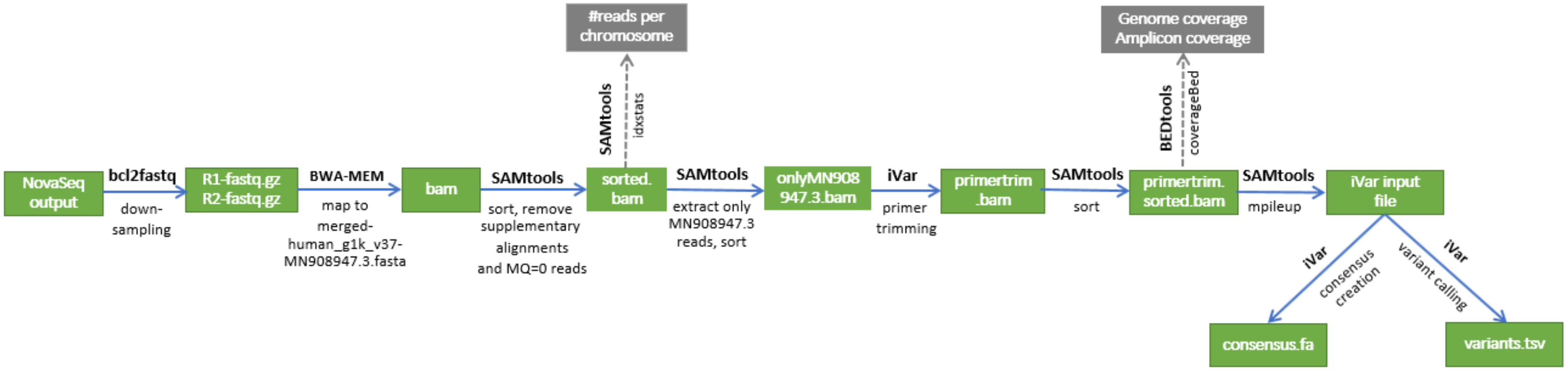

Figure S2. **Bioinformatics pipeline for the analysis of SARS-CoV-2 tiled amplicon Illumina sequencing data.**

Following mapping with "bwa mem", "samtools view" is run with the parameters -q 1 to filter out multi-mapping reads and with -F 2048 to filter out supplementary alignment reads. "samtools mpileup" is run with the iVar-recommended parameters -A -B -d 600000 -Q 0. To generate a PCR primer-trimmed bam file, the iVar program is used to trim the ARTIC V3 primers with "ivar trim -b <primers.bed>". For Illumina MiSeq runs, iVar is used for consensus sequence generation with the minimum depth set to 10 reads (-m 10), minimum quality value 10 (-q 10) and minimum variant frequency threshold of 50% (-t 0.5). To create consensus sequence for Illumina NovaSeq runs, we use a dynamic read depth cutoff (-m) as described in the Methods section. "samtools idxstats" is used to calculate the number of reads mapped to the viral and human reference sequence chromosome. To calculate minimum and average amplicon coverage from the primer-trimmed bam file, the "bedtools coverage" command is used from the BEDtools package.

Table S1. Primer pools for the automated, high-throughput SARS-CoV-2 NGS workflow

Primer Name (nCoV-2019_)	Sequence (5' to 3')	Pool	Ratio[1]
1_LEFT	ACACTCTTTCCCTACACGACGCTCTTCCGATCT ACCAACCAACTTTCGATCTCTTGT	1A	1X
**1_RIGHTv2**	**GTGACTGGAGTTCAGACGTGTGCTCTTCCGATCT AAGTGCCATCTTTAAGATGTTGACG**	1A	1X
2_LEFT	ACACTCTTTCCCTACACGACGCTCTTCCGATCT CTGTTTTACAGGTTCGCGACGT	2A	1X
2_RIGHT	GTGACTGGAGTTCAGACGTGTGCTCTTCCGATCT TAAGGATCAGTGCCAAGCTCGT	2A	1X
3_LEFT	ACACTCTTTCCCTACACGACGCTCTTCCGATCT CGGTAATAAAGGAGCTGGTGGC	1B	1X
3_RIGHT	GTGACTGGAGTTCAGACGTGTGCTCTTCCGATCT AAGGTGTCTGCAATTCATAGCTCT	1B	1X
4_LEFT	ACACTCTTTCCCTACACGACGCTCTTCCGATCT GGTGTATACTGCTGCCGTGAAC	2B	1X
4_RIGHT	GTGACTGGAGTTCAGACGTGTGCTCTTCCGATCT CACAAGTAGTGGCACCTTCTTTAGT	2B	1X
5_LEFT	ACACTCTTTCCCTACACGACGCTCTTCCGATCT TGGTGAAACTTCATGGCAGACG	1A	1X
5_RIGHT	GTGACTGGAGTTCAGACGTGTGCTCTTCCGATCT ATTGATGTTGACTTTCTCTTTTTGGAGT	1A	1X
6_LEFT	ACACTCTTTCCCTACACGACGCTCTTCCGATCT GGTGTTGTTGGAGAAGGTTCCG	2A	1X
6_RIGHT	GTGACTGGAGTTCAGACGTGTGCTCTTCCGATCT TAGCGGCCTTCTGTAAAACACG	2A	1X
7_LEFT	ACACTCTTTCCCTACACGACGCTCTTCCGATCT ATCAGAGGCTGCTCGTGTTGTA	1B	1X
7_LEFT_alt0	ACACTCTTTCCCTACACGACGCTCTTCCGATCT CATTTGCATCAGAGGCTGCTCG	1A	1X
7_RIGHT	GTGACTGGAGTTCAGACGTGTGCTCTTCCGATCT TGCACAGGTGACAATTTGTCCA	1B	1X
7_RIGHT_alt5	GTGACTGGAGTTCAGACGTGTGCTCTTCCGATCT AGGTGACAATTTGTCCACCGAC	1A	1X
8_LEFT	ACACTCTTTCCCTACACGACGCTCTTCCGATCT AGAGTTTCTTAGAGACGGTTGGGA	2B	1X
8_RIGHT	GTGACTGGAGTTCAGACGTGTGCTCTTCCGATCT GCTTCAACAGCTTCACTAGTAGGT	2B	1X
9_LEFT	ACACTCTTTCCCTACACGACGCTCTTCCGATCT TCCCACAGAAGTGTTAACAGAGGA	1B	1X
9_LEFT_alt4	ACACTCTTTCCCTACACGACGCTCTTCCGATCT TTCCCACAGAAGTGTTAACAGAGG	1A	1X
**9_RIGHTv2**	**GTGACTGGAGTTCAGACGTGTGCTCTTCCGATCT TTTATGACAGCATCTGCCACAA**	1B	1X
9_RIGHT_alt2	GTGACTGGAGTTCAGACGTGTGCTCTTCCGATCT GACAGCATCTGCCACAACACAG	1A	1X
10_LEFT	ACACTCTTTCCCTACACGACGCTCTTCCGATCT TGAGAAGTGCTCTGCCTATACAGT	2A	1X
10_RIGHT	GTGACTGGAGTTCAGACGTGTGCTCTTCCGATCT TCATCTAACCAATCTTCTTCTTGCTCT	2A	1X
11_LEFT	ACACTCTTTCCCTACACGACGCTCTTCCGATCT GGAATTTGGTGCCACTTCTGCT	1B	1X
11_RIGHT	GTGACTGGAGTTCAGACGTGTGCTCTTCCGATCT TCATCAGATTCAACTTGCATGGCA	1B	1X
12_LEFT	ACACTCTTTCCCTACACGACGCTCTTCCGATCT AAACATGGAGGAGGTGTTGCAG	2B	1X
12_RIGHT	GTGACTGGAGTTCAGACGTGTGCTCTTCCGATCT TTCACTCTTCATTTCCAAAAAGCTTGA	2B	1X
13_LEFT	ACACTCTTTCCCTACACGACGCTCTTCCGATCT TCGCACAAATGTCTACTTAGCTGT	1A	1X
**13_RIGHTv2**	**GTGACTGGAGTTCAGACGTGTGCTCTTCCGATCT ATAACCACAGCAGTTAAAACAC**	1A	1X
14_LEFT	ACACTCTTTCCCTACACGACGCTCTTCCGATCT CATCCAGATTCTGCCACTCTTGT	2A	1X
14_LEFT_alt4	ACACTCTTTCCCTACACGACGCTCTTCCGATCT TGGCAATCTTCATCCAGATTCTGC	2B	1X
14_RIGHT	GTGACTGGAGTTCAGACGTGTGCTCTTCCGATCT AGTTTCCACACAGACAGGCATT	2A	1X
14_RIGHT_alt2	GTGACTGGAGTTCAGACGTGTGCTCTTCCGATCT TGCGTGTTTCTTCTGCATGTGC	2B	1X
15_LEFT	ACACTCTTTCCCTACACGACGCTCTTCCGATCT ACAGTGCTTAAAAAGTGTAAAAGTGCC	1B	1X
15_LEFT_alt1	ACACTCTTTCCCTACACGACGCTCTTCCGATCT AGTGCTTAAAAAGTGTAAAAGTGCCT	1A	1X
**15_RIGHTv2**	**GTGACTGGAGTTCAGACGTGTGCTCTTCCGATCT AGAAACAGAAACTGTAGCTGGC**	1B	1X
15_RIGHT_alt3	GTGACTGGAGTTCAGACGTGTGCTCTTCCGATCT ACTGTAGCTGGCACTTTGAGAGA	1A	1X
16_LEFT	ACACTCTTTCCCTACACGACGCTCTTCCGATCT AATTTGGAAGAAGCTGCTCGGT	2A	1X
16_RIGHT	GTGACTGGAGTTCAGACGTGTGCTCTTCCGATCT CACAACTTGCGTGTGGAGGTTA	2A	1X
17_LEFT	ACACTCTTTCCCTACACGACGCTCTTCCGATCT CTTCTTTCTTTGAGAGAAGTGAGGACT	1B	1X
17_RIGHT	GTGACTGGAGTTCAGACGTGTGCTCTTCCGATCT TTTGTTGGAGTGTTAACAATGCAGT	1B	1X
18_LEFT	ACACTCTTTCCCTACACGACGCTCTTCCGATCT TGGAAATACCCACAAGTTAATGGTTTAAC	2B	1X
18_LEFT_alt2	ACACTCTTTCCCTACACGACGCTCTTCCGATCT ACTTCTATTAAATGGGCAGATAACAACTGT	2A	1X

18_RIGHT	GTGACTGGAGTTCAGACGTGTGCTCTTCCGATCT AGCTTGTTTACCACACGTACAAGG	2B	1X
18_RIGHT_alt1	GTGACTGGAGTTCAGACGTGTGCTCTTCCGATCT GCTTGTTTACCACACGTACAAGG	2A	1X
19_LEFT	ACACTCTTTCCCTACACGACGCTCTTCCGATCT GCTGTTATGTACATGGGCACACT	1A	1X
19_RIGHT	GTGACTGGAGTTCAGACGTGTGCTCTTCCGATCT TGTCCAACTTAGGGTCAATTTCTGT	1A	1X
20_LEFT	ACACTCTTTCCCTACACGACGCTCTTCCGATCT ACAAAGAAAACAGTTACACAACAACCA	2B	1X
20_RIGHT	GTGACTGGAGTTCAGACGTGTGCTCTTCCGATCT ACGTGGCTTTATTAGTTGCATTGTT	2B	1X
**21_LEFTv2**	**ACACTCTTTCCCTACACGACGCTCTTCCGATCT TGGTGGCTATTGATTATAAACACTACACA**	1B	1X
21_LEFT_alt2	ACACTCTTTCCCTACACGACGCTCTTCCGATCT GGCTATTGATTATAAACACTACACACCCT	1A	1X
21_RIGHT	GTGACTGGAGTTCAGACGTGTGCTCTTCCGATCT TAGATCTGTGTGGCCAACCTCT	1B	1X
21_RIGHT_alt0	GTGACTGGAGTTCAGACGTGTGCTCTTCCGATCT GATCTGTGTGGCCAACCTCTTC	1A	1X
22_LEFT	ACACTCTTTCCCTACACGACGCTCTTCCGATCT ACTACCGAAGTTGTAGGAGACATTATACT	2A	1X
22_RIGHT	GTGACTGGAGTTCAGACGTGTGCTCTTCCGATCT ACAGTATTCTTTGCTATAGTAGTCGGC	2A	1X
23_LEFT	ACACTCTTTCCCTACACGACGCTCTTCCGATCT ACAACTACTAACATAGTTACACGGTGT	1B	1X
23_RIGHT	GTGACTGGAGTTCAGACGTGTGCTCTTCCGATCT ACCAGTACAGTAGGTTGCAATAGTG	1B	1X
24_LEFT	ACACTCTTTCCCTACACGACGCTCTTCCGATCT AGGCATGCCTTCTTACTGTACTG	2B	1X
24_RIGHT	GTGACTGGAGTTCAGACGTGTGCTCTTCCGATCT ACATTCTAACCATAGCTGAAATCGGG	2B	1X
25_LEFT	ACACTCTTTCCCTACACGACGCTCTTCCGATCT GCAATTGTTTTTCAGCTATTTTGCAGT	1A	1X
25_RIGHT	GTGACTGGAGTTCAGACGTGTGCTCTTCCGATCT ACTGTAGTGACAAGTCTCTCGCA	1A	1X
26_LEFT	ACACTCTTTCCCTACACGACGCTCTTCCGATCT TTGTGATACATTCTGTGCTGGTAGT	2A, 2B	1X
26_RIGHT	GTGACTGGAGTTCAGACGTGTGCTCTTCCGATCT TCCGCACTATCACCAACATCAG	2A, 2B	1X
27_LEFT	ACACTCTTTCCCTACACGACGCTCTTCCGATCT ACTACAGTCAGCTTATGTGTCAACC	1B	1X
27_RIGHT	GTGACTGGAGTTCAGACGTGTGCTCTTCCGATCT AATACAAGCACCAAGGTCACGG	1B	1X
28_LEFT	ACACTCTTTCCCTACACGACGCTCTTCCGATCT ACATAGAAGTTACTGGCGATAGTTGT	2B	1X
28_RIGHT	GTGACTGGAGTTCAGACGTGTGCTCTTCCGATCT TGTTTAGACATGACATGAACAGGTGT	2B	1X
29_LEFT	ACACTCTTTCCCTACACGACGCTCTTCCGATCT ACTTGTGTTCCTTTTTGTTGCTGC	1A, 1B	1X
29_RIGHT	GTGACTGGAGTTCAGACGTGTGCTCTTCCGATCT AGTGTACTCTATAAGTTTTGATGGTGTGT	1A, 1B	1X
30_LEFT	ACACTCTTTCCCTACACGACGCTCTTCCGATCT GCACAACTAATGGTGACTTTTTGCA	2A	1X
30_RIGHT	GTGACTGGAGTTCAGACGTGTGCTCTTCCGATCT ACCACTAGTAGATACACAAACACCAG	2A	1X
31_LEFT	ACACTCTTTCCCTACACGACGCTCTTCCGATCT TTCTGAGTACTGTAGGCACGGC	1B	1X
31_RIGHT	GTGACTGGAGTTCAGACGTGTGCTCTTCCGATCT ACAGAATAAACACCAGGTAAGAATGAGT	1B	1X
32_LEFT	ACACTCTTTCCCTACACGACGCTCTTCCGATCT TGGTGAATACAGTCATGTAGTTGCC	2B	1X
**32_RIGHTv2**	**GTGACTGGAGTTCAGACGTGTGCTCTTCCGATCT GGTAATAGCACATCACTACGCAAC**	2B	1X
33_LEFT	ACACTCTTTCCCTACACGACGCTCTTCCGATCT ACTTTTGAAGAAGCTGCGCTGT	1A	1X
33_RIGHT	GTGACTGGAGTTCAGACGTGTGCTCTTCCGATCT TGGACAGTAAACTACGTCATCAAGC	1A	1X
34_LEFT	ACACTCTTTCCCTACACGACGCTCTTCCGATCT TCCCATCTGGTAAAGTTGAGGGT	2A	1X
34_RIGHT	GTGACTGGAGTTCAGACGTGTGCTCTTCCGATCT AGTGAAATTGGGCCTCATAGCA	2A	1X
35_LEFT	ACACTCTTTCCCTACACGACGCTCTTCCGATCT TGTTCGCATTCAACCAGGACAG	1B	1X
35_RIGHT	GTGACTGGAGTTCAGACGTGTGCTCTTCCGATCT ACTTCATAGCCACAAGGTTAAAGTCA	1B	1X
**36_LEFTv2**	**ACACTCTTTCCCTACACGACGCTCTTCCGATCT ATTACAGTTAATGTTTTAGCTTGGTTGTA**	2B	1X
36_RIGHT	GTGACTGGAGTTCAGACGTGTGCTCTTCCGATCT GAACAAAGACCATTGAGTACTCTGGA	2B	1X
37_LEFT	ACACTCTTTCCCTACACGACGCTCTTCCGATCT ACACACCACTGGTTGTTACTCAC	1A	1X
37_RIGHT	GTGACTGGAGTTCAGACGTGTGCTCTTCCGATCT GTCCACACTCTCCTAGCACCAT	1A	1X
38_LEFT	ACACTCTTTCCCTACACGACGCTCTTCCGATCT ACTGTGTTATGTATGCATCAGCTGT	2A	1X
**38_RIGHTv2**	**GTGACTGGAGTTCAGACGTGTGCTCTTCCGATCT ATCATAAACACCAAGAGTCAGTCTAAA**	2A	1X
39_LEFT	ACACTCTTTCCCTACACGACGCTCTTCCGATCT AGTATTGCCCTATTTTCTTCATAACTGGT	1B	1X
39_RIGHT	GTGACTGGAGTTCAGACGTGTGCTCTTCCGATCT TGTAACTGGACACATTGAGCCC	1B	1X
40_LEFT	ACACTCTTTCCCTACACGACGCTCTTCCGATCT TGCACATCAGTAGTCTTACTCTCAGT	2B	1X
40_RIGHT	GTGACTGGAGTTCAGACGTGTGCTCTTCCGATCT CATGGCTGCATCACGGTCAAAT	2B	1X
41_LEFT	ACACTCTTTCCCTACACGACGCTCTTCCGATCT GTTCCCTTCCATCATATGCAGCT	1A	1X
41_RIGHT	GTGACTGGAGTTCAGACGTGTGCTCTTCCGATCT TGGTATGACAACCATTAGTTTGGCT	1A	1X

42_LEFT	ACACTCTTTCCCTACACGACGCTCTTCCGATCT TGCAAGAGATGGTTGTGTTCCC	2A	1X
42_RIGHT	GTGACTGGAGTTCAGACGTGTGCTCTTCCGATCT CCTACCTCCCTTTGTTGTGTTGT	2A	1X
43_LEFT	ACACTCTTTCCCTACACGACGCTCTTCCGATCT TACGACAGATGTCTTGTGCTGC	1B	1X
43_RIGHT	GTGACTGGAGTTCAGACGTGTGCTCTTCCGATCT AGCAGCATCTACAGCAAAAGCA	1B	1X
44_LEFT	ACACTCTTTCCCTACACGACGCTCTTCCGATCT TGCCACAGTACGTCTACAAGCT	2B	1X
44_LEFT_alt3	ACACTCTTTCCCTACACGACGCTCTTCCGATCT CCACAGTACGTCTACAAGCTGG	2A	1X
44_RIGHT	GTGACTGGAGTTCAGACGTGTGCTCTTCCGATCT AACCTTTCCACATACCGCAGAC	2B	1X
44_RIGHT_alt0	GTGACTGGAGTTCAGACGTGTGCTCTTCCGATCT CGCAGACGGTACAGACTGTGTT	2A	1X
45_LEFT	ACACTCTTTCCCTACACGACGCTCTTCCGATCT TACCTACAACTTGTGCTAATGACCC	1A	1X
45_LEFT_alt2	ACACTCTTTCCCTACACGACGCTCTTCCGATCT AGTATGTACAAATACCTACAACTTGTGCT	1B	1X
45_RIGHT	GTGACTGGAGTTCAGACGTGTGCTCTTCCGATCT AAATTGTTTCTTCATGTTGGTAGTTAGAGA	1A	1X
45_RIGHT_alt7	GTGACTGGAGTTCAGACGTGTGCTCTTCCGATCT TTCATGTTGGTAGTTAGAGAAAGTGTGTC	1B	1X
46_LEFT	ACACTCTTTCCCTACACGACGCTCTTCCGATCT TGTCGCTTCCAAGAAAAGGACG	2B	1X
46_LEFT_alt1	ACACTCTTTCCCTACACGACGCTCTTCCGATCT CGCTTCCAAGAAAAGGACGAAGA	2A	1X
46_RIGHT	GTGACTGGAGTTCAGACGTGTGCTCTTCCGATCT CACGTTCACCTAAGTTGGCGTA	2B	1X
46_RIGHT_alt2	GTGACTGGAGTTCAGACGTGTGCTCTTCCGATCT CACGTTCACCTAAGTTGGCGTAT	2A	1X
47_LEFT	ACACTCTTTCCCTACACGACGCTCTTCCGATCT AGGACTGGTATGATTTTGTAGAAAACCC	1A	1X
47_RIGHT	GTGACTGGAGTTCAGACGTGTGCTCTTCCGATCT AATAACGGTCAAAGAGTTTTAACCTCTC	1A	1X
48_LEFT	ACACTCTTTCCCTACACGACGCTCTTCCGATCT TGTTGACACTGACTTAACAAAGCCT	2B	1X
48_RIGHT	GTGACTGGAGTTCAGACGTGTGCTCTTCCGATCT TAGATTACCAGAAGCAGCGTGC	2B	1X
49_LEFT	ACACTCTTTCCCTACACGACGCTCTTCCGATCT AGGAATTACTTGTGTATGCTGCTGA	1B	1X
49_RIGHT	GTGACTGGAGTTCAGACGTGTGCTCTTCCGATCT TGACGATGACTTGGTTAGCATTAATACA	1B	1X
50_LEFT	ACACTCTTTCCCTACACGACGCTCTTCCGATCT GTTGATAAGTACTTTGATTGTTACGATGGT	2A	1X
50_RIGHT	GTGACTGGAGTTCAGACGTGTGCTCTTCCGATCT TAACATGTTGTGCCAACCACCA	2A	1X
51_LEFT	ACACTCTTTCCCTACACGACGCTCTTCCGATCT TCAATAGCCGCCACTAGAGGAG	1A	1X
51_RIGHT	GTGACTGGAGTTCAGACGTGTGCTCTTCCGATCT AGTGCATTAACATTGGCCGTGA	1A	1X
52_LEFT	ACACTCTTTCCCTACACGACGCTCTTCCGATCT CATCAGGAGATGCCACAACTGC	2B	1X
52_RIGHT	GTGACTGGAGTTCAGACGTGTGCTCTTCCGATCT GTTGAGAGCAAAATTCATGAGGTCC	2B	1X
53_LEFT	ACACTCTTTCCCTACACGACGCTCTTCCGATCT AGCAAAATGTTGGACTGAGACTGA	1B	1X
53_RIGHT	GTGACTGGAGTTCAGACGTGTGCTCTTCCGATCT AGCCTCATAAAACTCAGGTTCCC	1B	1X
54_LEFT	ACACTCTTTCCCTACACGACGCTCTTCCGATCT TGAGTTAACAGGACACATGTTAGACA	2A	1X
54_RIGHT	GTGACTGGAGTTCAGACGTGTGCTCTTCCGATCT AACCAAAAACTTGTCCATTAGCACA	2A	1X
55_LEFT	ACACTCTTTCCCTACACGACGCTCTTCCGATCT ACTCAACTTTACTTAGGAGGTATGAGCT	1A	1X
55_RIGHT	GTGACTGGAGTTCAGACGTGTGCTCTTCCGATCT GGTGTACTCTCCTATTTGTACTTTACTGT	1A	1X
56_LEFT	ACACTCTTTCCCTACACGACGCTCTTCCGATCT ACCTAGACCACCACTTAACCGA	2B	1X
56_RIGHT	GTGACTGGAGTTCAGACGTGTGCTCTTCCGATCT ACACTATGCGAGCAGAAGGGTA	2B	1X
57_LEFT	ACACTCTTTCCCTACACGACGCTCTTCCGATCT ATTCTACACTCCAGGGACCACC	1B	1X
57_RIGHT	GTGACTGGAGTTCAGACGTGTGCTCTTCCGATCT GTAATTGAGCAGGGTCGCCAAT	1B	1X
58_LEFT	ACACTCTTTCCCTACACGACGCTCTTCCGATCT TGATTTGAGTGTTGTCAATGCCAGA	2A	1X
58_RIGHT	GTGACTGGAGTTCAGACGTGTGCTCTTCCGATCT CTTTTCTCCAAGCAGGGTTACGT	2A	1X
59_LEFT	ACACTCTTTCCCTACACGACGCTCTTCCGATCT TCACGCATGATGTTTCATCTGCA	1A	1X
59_RIGHT	GTGACTGGAGTTCAGACGTGTGCTCTTCCGATCT AAGAGTCCTGTTACATTTTCAGCTTG	1A	1X
60_LEFT	ACACTCTTTCCCTACACGACGCTCTTCCGATCT TGATAGAGACCTTTATGACAAGTTGCA	2B	1X
60_RIGHT	GTGACTGGAGTTCAGACGTGTGCTCTTCCGATCT GGTACCAACAGCTTCTCTAGTAGC	2B	1X
61_LEFT	ACACTCTTTCCCTACACGACGCTCTTCCGATCT TGTTTATCACCCGCGAAGAAGC	1B	1X
61_RIGHT	GTGACTGGAGTTCAGACGTGTGCTCTTCCGATCT ATCACATAGACAACAGGTGCGC	1B	1X
62_LEFT	ACACTCTTTCCCTACACGACGCTCTTCCGATCT GGCACATGGCTTTGAGTTGACA	2A	1X
62_RIGHT	GTGACTGGAGTTCAGACGTGTGCTCTTCCGATCT GTTGAACCTTTCTACAAGCCGC	2A	1X
63_LEFT	ACACTCTTTCCCTACACGACGCTCTTCCGATCT TGTTAAGCGTGTTGACTGGACT	1A	1X
63_RIGHT	GTGACTGGAGTTCAGACGTGTGCTCTTCCGATCT ACAAACTGCCACCATCACAACC	1A	1X

64_LEFT	ACACTCTTTCCCTACACGACGCTCTTCCGATCT TCGATAGATATCCTGCTAATTCCATTGT	2B	1X
64_RIGHT	GTGACTGGAGTTCAGACGTGTGCTCTTCCGATCT AGTCTTGTAAAAGTGTTCCAGAGGT	2B	1X
65_LEFT	ACACTCTTTCCCTACACGACGCTCTTCCGATCT GCTGGCTTTAGCTTGTGGGTTT	1B	1X
65_RIGHT	GTGACTGGAGTTCAGACGTGTGCTCTTCCGATCT TGTCAGTCATAGAACAAACACCAATAGT	1B	1X
66_LEFT	ACACTCTTTCCCTACACGACGCTCTTCCGATCT GGGTGTGGACATTGCTGCTAAT	2A	1X
66_RIGHT	GTGACTGGAGTTCAGACGTGTGCTCTTCCGATCT TCAATTTCCATTTGACTCCTGGGT	2A	1X
67_LEFT	ACACTCTTTCCCTACACGACGCTCTTCCGATCT GTTGTCCAACAATTACCTGAAACTTACT	1A, 1B	1X
67_RIGHT	GTGACTGGAGTTCAGACGTGTGCTCTTCCGATCT CAACCTTAGAAACTACAGATAAATCTTGGG	1A, 1B	1X
68_LEFT	ACACTCTTTCCCTACACGACGCTCTTCCGATCT ACAGGTTCATCTAAGTGTGTGTGT	2A, 2B	1X
68_RIGHT	GTGACTGGAGTTCAGACGTGTGCTCTTCCGATCT CTCCTTTATCAGAACCAGCACCA	2A, 2B	1X
69_LEFT	ACACTCTTTCCCTACACGACGCTCTTCCGATCT TGTCGCAAAATATACTCAACTGTGTCA	1B	1X
69_RIGHT	GTGACTGGAGTTCAGACGTGTGCTCTTCCGATCT TCTTTATAGCCACGGAACCTCCA	1B	1X
70_LEFT	ACACTCTTTCCCTACACGACGCTCTTCCGATCT ACAAAAGAAAATGACTCTAAAGAGGGTTT	2B	1X
70_RIGHT	GTGACTGGAGTTCAGACGTGTGCTCTTCCGATCT TGACCTTCTTTTAAAGACATAACAGCAG	2B	1X
71_LEFT	ACACTCTTTCCCTACACGACGCTCTTCCGATCT ACAAATCCAATTCAGTTGTCTTCCTATTC	1A	1X
71_RIGHT	GTGACTGGAGTTCAGACGTGTGCTCTTCCGATCT TGGAAAAGAAAGGTAAGAACAAGTCCT	1A	1X
72_LEFT	ACACTCTTTCCCTACACGACGCTCTTCCGATCT ACACGTGGTGTTTATTACCCTGAC	2B	1X
72_RIGHT	GTGACTGGAGTTCAGACGTGTGCTCTTCCGATCT ACTCTGAACTCACTTTCCATCCAAC	2B	1X
73_LEFT	ACACTCTTTCCCTACACGACGCTCTTCCGATCT CAATTTTGTAATGATCCATTTTTGGGTGT	1B	1X
73_RIGHT	GTGACTGGAGTTCAGACGTGTGCTCTTCCGATCT CACCAGCTGTCCAACCTGAAGA	1B	1X
74_LEFT	ACACTCTTTCCCTACACGACGCTCTTCCGATCT ACATCACTAGGTTTCAAACTTTACTTGC	2A, 2B	2X
74_RIGHT	GTGACTGGAGTTCAGACGTGTGCTCTTCCGATCT GCAACACAGTTGCTGATTCTCTTC	2A, 2B	2X
75_LEFT	ACACTCTTTCCCTACACGACGCTCTTCCGATCT AGAGTCCAACCAACAGAATCTATTGT	1A, 1B	1X
75_RIGHT	GTGACTGGAGTTCAGACGTGTGCTCTTCCGATCT ACCACCAACCTTAGAATCAAGATTGT	1A, 1B	1X
76_LEFT	ACACTCTTTCCCTACACGACGCTCTTCCGATCT AGGGCAAACTGGAAAGATTGCT	2B	1X
76_LEFT_alt3	ACACTCTTTCCCTACACGACGCTCTTCCGATCT GGGCAAACTGGAAAGATTGCTGA	2A	1X
**76_RIGHTv2**	**GTGACTGGAGTTCAGACGTGTGCTCTTCCGATCT TCTCTGCCAAATTGTTGGAAAGGCA**	2B	1X
76_RIGHT_alt0	GTGACTGGAGTTCAGACGTGTGCTCTTCCGATCT ACCTGTGCCTGTTAAACCATTGA	2A	1X
77_LEFT	ACACTCTTTCCCTACACGACGCTCTTCCGATCT CCAGCAACTGTTTGTGGACCTA	1B	1X
77_RIGHT	GTGACTGGAGTTCAGACGTGTGCTCTTCCGATCT CAGCCCCTATTAAACAGCCTGC	1B	1X
78_LEFT	ACACTCTTTCCCTACACGACGCTCTTCCGATCT CAACTTACTCCTACTTGGCGTGT	2B	1X
78_RIGHT	GTGACTGGAGTTCAGACGTGTGCTCTTCCGATCT TGTGTACAAAAACTGCCATATTGCA	2B	1X
79_LEFT	ACACTCTTTCCCTACACGACGCTCTTCCGATCT GTGGTGATTCAACTGAATGCAGC	1A, 1B	1X
79_RIGHT	GTGACTGGAGTTCAGACGTGTGCTCTTCCGATCT CATTTCATCTGTGAGCAAAGGTGG	1A, 1B	1X
80_LEFT	ACACTCTTTCCCTACACGACGCTCTTCCGATCT TTGCCTTGGTGATATTGCTGCT	2A	1X
80_RIGHT	GTGACTGGAGTTCAGACGTGTGCTCTTCCGATCT TGGAGCTAAGTTGTTTAACAAGCG	2A	1X
81_LEFT	ACACTCTTTCCCTACACGACGCTCTTCCGATCT GCACTTGGAAAACTTCAAGATGTGG	1B	1X
81_RIGHT	GTGACTGGAGTTCAGACGTGTGCTCTTCCGATCT GTGAAGTTCTTTTCTTGTGCAGGG	1B	1X
82_LEFT	ACACTCTTTCCCTACACGACGCTCTTCCGATCT GGGCTATCATCTTATGTCCTTCCCT	2B	1X
82_RIGHT	GTGACTGGAGTTCAGACGTGTGCTCTTCCGATCT TGCCAGAGATGTCACCTAAATCAA	2B	1X
83_LEFT	ACACTCTTTCCCTACACGACGCTCTTCCGATCT TCCTTTGCAACCTGAATTAGACTCA	1A	1X
83_RIGHT	GTGACTGGAGTTCAGACGTGTGCTCTTCCGATCT TTTGACTCCTTTGAGCACTGGC	1A	1X
84_LEFT	ACACTCTTTCCCTACACGACGCTCTTCCGATCT TGCTGTAGTTGTCTCAAGGGCT	2A	1X
84_RIGHT	GTGACTGGAGTTCAGACGTGTGCTCTTCCGATCT AGGTGTGAGTAAACTGTTACAAACAAC	2A	1X
85_LEFT	ACACTCTTTCCCTACACGACGCTCTTCCGATCT ACTAGCACTCTCCAAGGGTGTT	1B	1X
85_RIGHT	GTGACTGGAGTTCAGACGTGTGCTCTTCCGATCT ACACAGTCTTTTACTCCAGATTCCC	1B	1X
86_LEFT	ACACTCTTTCCCTACACGACGCTCTTCCGATCT TCAGGTGATGGCACAACAAGTC	2B	1X
86_RIGHT	GTGACTGGAGTTCAGACGTGTGCTCTTCCGATCT ACGAAAGCAAGAAAAAGAAGTACGC	2B	1X
87_LEFT	ACACTCTTTCCCTACACGACGCTCTTCCGATCT CGACTACTAGCGTGCCTTTGTA	1A	1X
87_RIGHT	GTGACTGGAGTTCAGACGTGTGCTCTTCCGATCT ACTAGGTTCCATTGTTCAAGGAGC	1A	1X

88_LEFT	ACACTCTTTCCCTACACGACGCTCTTCCGATCT CCATGGCAGATTCCAACGGTAC	2A	1X
88_RIGHT	GTGACTGGAGTTCAGACGTGTGCTCTTCCGATCT TGGTCAGAATAGTGCCATGGAGT	2A	1X
**89_LEFTv2**	**ACACTCTTTCCCTACACGACGCTCTTCCGATCT CGCGTACGCGTTCCATGTGGTC**	1B	1X
89_LEFT_alt2	ACACTCTTTCCCTACACGACGCTCTTCCGATCT CGCGTTCCATGTGGTCATTCAA	1A	1X
**89_RIGHTv2**	**GTGACTGGAGTTCAGACGTGTGCTCTTCCGATCT ATAGTAACCTGAAAGTCAACGAGAT**	1B	1X
89_RIGHT_alt4	GTGACTGGAGTTCAGACGTGTGCTCTTCCGATCT ACGAGATGAAACATCTGTTGTCACT	1A	1X
90_LEFT	ACACTCTTTCCCTACACGACGCTCTTCCGATCT ACACAGACCATTCCAGTAGCAGT	2B	1X
90_RIGHT	GTGACTGGAGTTCAGACGTGTGCTCTTCCGATCT TGAAATGGTGAATTGCCCTCGT	2B	1X
91_LEFT	ACACTCTTTCCCTACACGACGCTCTTCCGATCT TCACTACCAAGAGTGTGTTAGAGGT	1B	1X
91_RIGHT	GTGACTGGAGTTCAGACGTGTGCTCTTCCGATCT TTCAAGTGAGAACCAAAAGATAATAAGCA	1B	1X
92_LEFT	ACACTCTTTCCCTACACGACGCTCTTCCGATCT TTTGTGCTTTTTAGCCTTTCTGCT	2A	1X
92_RIGHT	GTGACTGGAGTTCAGACGTGTGCTCTTCCGATCT AGGTTCCTGGCAATTAATTGTAAAAGG	2A	1X
93_LEFT	ACACTCTTTCCCTACACGACGCTCTTCCGATCT TGAGGCTGGTTCTAAATCACCCA	1A	1X
93_RIGHT	GTGACTGGAGTTCAGACGTGTGCTCTTCCGATCT AGGTCTTCCTTGCCATGTTGAG	1A	1X
94_LEFT	ACACTCTTTCCCTACACGACGCTCTTCCGATCT GGCCCCAAGGTTTACCCAATAA	2B	1X
94_RIGHT	GTGACTGGAGTTCAGACGTGTGCTCTTCCGATCT TTTGGCAATGTTGTTCCTTGAGG	2B	1X
95_LEFT	ACACTCTTTCCCTACACGACGCTCTTCCGATCT TGAGGGAGCCTTGAATACACCA	1B	1X
95_RIGHT	GTGACTGGAGTTCAGACGTGTGCTCTTCCGATCT CAGTACGTTTTTGCCGAGGCTT	1B	1X
96_LEFT	ACACTCTTTCCCTACACGACGCTCTTCCGATCT GCCAACAACAACAAGGCCAAAC	2A	1X
96_RIGHT	GTGACTGGAGTTCAGACGTGTGCTCTTCCGATCT TAGGCTCTGTTGGTGGGAATGT	2A	1X
97_LEFT	ACACTCTTTCCCTACACGACGCTCTTCCGATCT TGGATGACAAAGATCCAAATTTCAAAGA	1A	1X
97_RIGHT	GTGACTGGAGTTCAGACGTGTGCTCTTCCGATCT ACACACTGATTAAAGATTGCTATGTGAG	1A	1X
98_LEFT	ACACTCTTTCCCTACACGACGCTCTTCCGATCT AACAATTGCAACAATCCATGAGCA	2B	1X
98_RIGHT	GTGACTGGAGTTCAGACGTGTGCTCTTCCGATCT TTCTCCTAAGAAGCTATTAAAATCACATGG	2B	1X

[1] Relative primer concentration in each pool is indicated under Ratio.

SARS-CoV-2 specific sequences are from the ARTICv3 primer set (Quick 2020).

Primers from Itokawa et al (2020) are marked in bold.

Quick J, Loman NJ. hCoV-2019/nCoV-2019 Version 3 Amplicon Set. March 24, 2020. Available: https://artic.network/resources/ncov/ncov-amplicon-v3.pdf.

Itokawa K, Sekizuka T, Hashino M, Tanaka R, Kuroda M (2020) Disentangling primer interactions improves SARS-CoV-2 genome sequencing by multiplex tiling PCR. PLoS ONE 15(9): e0239403.

Table S2. Modified primer concentration for each pool for a standard ARTIC v3 workflow

Primer Name (nCoV-2019_)	Sequence (5' to 3')	Pool	Ratio[1]
1_LEFT	ACCAACCAACTTTCGATCTCTTGT	1	1X
1_RIGHT	CATCTTTAAGATGTTGACGTGCCTC	1	1X
2_LEFT	CTGTTTTACAGGTTCGCGACGT	2	1X
2_RIGHT	TAAGGATCAGTGCCAAGCTCGT	2	1X
3_LEFT	CGGTAATAAAGGAGCTGGTGGC	1	1X
3_RIGHT	AAGGTGTCTGCAATTCATAGCTCT	1	1X
4_LEFT	GGTGTATACTGCTGCCGTGAAC	2	1X
4_RIGHT	CACAAGTAGTGGCACCTTCTTTAGT	2	1X
5_LEFT	TGGTGAAACTTCATGGCAGACG	1	7X
5_RIGHT	ATTGATGTTGACTTTCTCTTTTTGGAGT	1	7X
6_LEFT	GGTGTTGTTGGAGAAGGTTCCG	2	2.5X
6_RIGHT	TAGCGGCCTTCTGTAAAACACG	2	2.5X
7_LEFT	ATCAGAGGCTGCTCGTGTTGTA	1	1X
7_LEFT_alt0	CATTTGCATCAGAGGCTGCTCG	1	1X
7_RIGHT	TGCACAGGTGACAATTTGTCCA	1	1X
7_RIGHT_alt5	AGGTGACAATTTGTCCACCGAC	1	1X
8_LEFT	AGAGTTTCTTAGAGACGGTTGGGA	2	1X
8_RIGHT	GCTTCAACAGCTTCACTAGTAGGT	2	1X
9_LEFT	TCCCACAGAAGTGTTAACAGAGGA	1	1X
9_LEFT_alt4	TTCCCACAGAAGTGTTAACAGAGG	1	1X
9_RIGHT	ATGACAGCATCTGCCACAACAC	1	1X
9_RIGHT_alt2	GACAGCATCTGCCACAACACAG	1	1X
10_LEFT	TGAGAAGTGCTCTGCCTATACAGT	2	1X
10_RIGHT	TCATCTAACCAATCTTCTTCTTGCTCT	2	1X
11_LEFT	GGAATTTGGTGCCACTTCTGCT	1	1X
11_RIGHT	TCATCAGATTCAACTTGCATGGCA	1	1X
12_LEFT	AAACATGGAGGAGGTGTTGCAG	2	7X
12_RIGHT	TTCACTCTTCATTTCCAAAAAGCTTGA	2	7X
13_LEFT	TCGCACAAATGTCTACTTAGCTGT	1	7X
13_RIGHT	ACCACAGCAGTTAAAACACCCT	1	7X
14_LEFT	CATCCAGATTCTGCCACTCTTGT	2	1X
14_LEFT_alt4	TGGCAATCTTCATCCAGATTCTGC	2	1X
14_RIGHT	AGTTTCCACACAGACAGGCATT	2	1X
14_RIGHT_alt2	TGCGTGTTTCTTCTGCATGTGC	2	1X
15_LEFT	ACAGTGCTTAAAAAGTGTAAAAGTGCC	1	1X
15_LEFT_alt1	AGTGCTTAAAAAGTGTAAAAGTGCCT	1	1X
15_RIGHT	AACAGAAACTGTAGCTGGCACT	1	1X
15_RIGHT_alt3	ACTGTAGCTGGCACTTTGAGAGA	1	1X
16_LEFT	AATTTGGAAGAAGCTGCTCGGT	2	1X
16_RIGHT	CACAACTTGCGTGTGGAGGTTA	2	1X

17_LEFT	CTTCTTTCTTTGAGAGAAGTGAGGACT	1	7X
17_RIGHT	TTTGTTGGAGTGTTAACAATGCAGT	1	7X
18_LEFT	TGGAAATACCCACAAGTTAATGGTTTAAC	2	1X
18_LEFT_alt2	ACTTCTATTAAATGGGCAGATAACAACTGT	2	1X
18_RIGHT	AGCTTGTTTACCACACGTACAAGG	2	1X
18_RIGHT_alt1	GCTTGTTTACCACACGTACAAGG	2	1X
19_LEFT	GCTGTTATGTACATGGGCACACT	1	2.5X
19_RIGHT	TGTCCAACTTAGGGTCAATTTCTGT	1	2.5X
20_LEFT	ACAAAGAAAACAGTTACACAACAACCA	2	1X
20_RIGHT	ACGTGGCTTTATTAGTTGCATTGTT	2	1X
21_LEFT	TGGCTATTGATTATAAACACTACACACCC	1	7X
21_LEFT_alt2	GGCTATTGATTATAAACACTACACACCCT	1	7X
21_RIGHT	TAGATCTGTGTGGCCAACCTCT	1	7X
21_RIGHT_alt0	GATCTGTGTGGCCAACCTCTTC	1	7X
22_LEFT	ACTACCGAAGTTGTAGGAGACATTATACT	2	1X
22_RIGHT	ACAGTATTCTTTGCTATAGTAGTCGGC	2	1X
23_LEFT	ACAACTACTAACATAGTTACACGGTGT	1	7X
23_RIGHT	ACCAGTACAGTAGGTTGCAATAGTG	1	7X
24_LEFT	AGGCATGCCTTCTTACTGTACTG	2	1X
24_RIGHT	ACATTCTAACCATAGCTGAAATCGGG	2	1X
25_LEFT	GCAATTGTTTTTCAGCTATTTTGCAGT	1	1X
25_RIGHT	ACTGTAGTGACAAGTCTCTCGCA	1	1X
26_LEFT	TTGTGATACATTCTGTGCTGGTAGT	2	7X
26_RIGHT	TCCGCACTATCACCAACATCAG	2	7X
27_LEFT	ACTACAGTCAGCTTATGTGTCAACC	1	1X
27_RIGHT	AATACAAGCACCAAGGTCACGG	1	1X
28_LEFT	ACATAGAAGTTACTGGCGATAGTTGT	2	2.5X
28_RIGHT	TGTTTAGACATGACATGAACAGGTGT	2	2.5X
29_LEFT	ACTTGTGTTCCTTTTTGTTGCTGC	1	2.5X
29_RIGHT	AGTGTACTCTATAAGTTTTGATGGTGTGT	1	2.5X
30_LEFT	GCACAACTAATGGTGACTTTTTGCA	2	1X
30_RIGHT	ACCACTAGTAGATACACAAACACCAG	2	1X
31_LEFT	TTCTGAGTACTGTAGGCACGGC	1	1X
31_RIGHT	ACAGAATAAACACCAGGTAAGAATGAGT	1	1X
32_LEFT	TGGTGAATACAGTCATGTAGTTGCC	2	1X
32_RIGHT	AGCACATCACTACGCAACTTTAGA	2	1X
33_LEFT	ACTTTTGAAGAAGCTGCGCTGT	1	1X
33_RIGHT	TGGACAGTAAACTACGTCATCAAGC	1	1X
34_LEFT	TCCCATCTGGTAAAGTTGAGGGT	2	1X
34_RIGHT	AGTGAAATTGGGCCTCATAGCA	2	1X
35_LEFT	TGTTCGCATTCAACCAGGACAG	1	1X
35_RIGHT	ACTTCATAGCCACAAGGTTAAAGTCA	1	1X
36_LEFT	TTAGCTTGGTTGTACGCTGCTG	2	2.5X

36_RIGHT	GAACAAAGACCATTGAGTACTCTGGA	2	2.5X
37_LEFT	ACACACCACTGGTTGTTACTCAC	1	1X
37_RIGHT	GTCCACACTCTCCTAGCACCAT	1	1X
38_LEFT	ACTGTGTTATGTATGCATCAGCTGT	2	2.5X
38_RIGHT	CACCAAGAGTCAGTCTAAAGTAGCG	2	2.5X
39_LEFT	AGTATTGCCCTATTTTCTTCATAACTGGT	1	1X
39_RIGHT	TGTAACTGGACACATTGAGCCC	1	1X
40_LEFT	TGCACATCAGTAGTCTTACTCTCAGT	2	1X
40_RIGHT	CATGGCTGCATCACGGTCAAAT	2	1X
41_LEFT	GTTCCCTTCCATCATATGCAGCT	1	1X
41_RIGHT	TGGTATGACAACCATTAGTTTGGCT	1	1X
42_LEFT	TGCAAGAGATGGTTGTGTTCCC	2	1X
42_RIGHT	CCTACCTCCCTTTGTTGTGTTGT	2	1X
43_LEFT	TACGACAGATGTCTTGTGCTGC	1	1X
43_RIGHT	AGCAGCATCTACAGCAAAAGCA	1	1X
44_LEFT	TGCCACAGTACGTCTACAAGCT	2	1X
44_LEFT_alt3	CCACAGTACGTCTACAAGCTGG	2	1X
44_RIGHT	AACCTTTCCACATACCGCAGAC	2	1X
44_RIGHT_alt0	CGCAGACGGTACAGACTGTGTT	2	1X
45_LEFT	TACCTACAACTTGTGCTAATGACCC	1	1X
45_LEFT_alt2	AGTATGTACAAATACCTACAACTTGTGCT	1	1X
45_RIGHT	AAATTGTTTCTTCATGTTGGTAGTTAGAGA	1	1X
45_RIGHT_alt7	TTCATGTTGGTAGTTAGAGAAAGTGTGTC	1	1X
46_LEFT	TGTCGCTTCCAAGAAAAGGACG	2	1X
46_LEFT_alt1	CGCTTCCAAGAAAAGGACGAAGA	2	1X
46_RIGHT	CACGTTCACCTAAGTTGGCGTA	2	1X
46_RIGHT_alt2	CACGTTCACCTAAGTTGGCGTAT	2	1X
47_LEFT	AGGACTGGTATGATTTTGTAGAAAACCC	1	1X
47_RIGHT	AATAACGGTCAAAGAGTTTTAACCTCTC	1	1X
48_LEFT	TGTTGACACTGACTTAACAAAGCCT	2	1X
48_RIGHT	TAGATTACCAGAAGCAGCGTGC	2	1X
49_LEFT	AGGAATTACTTGTGTATGCTGCTGA	1	1X
49_RIGHT	TGACGATGACTTGGTTAGCATTAATACA	1	1X
50_LEFT	GTTGATAAGTACTTTGATTGTTACGATGGT	2	1X
50_RIGHT	TAACATGTTGTGCCAACCACCA	2	1X
51_LEFT	TCAATAGCCGCCACTAGAGGAG	1	1X
51_RIGHT	AGTGCATTAACATTGGCCGTGA	1	1X
52_LEFT	CATCAGGAGATGCCACAACTGC	2	1X
52_RIGHT	GTTGAGAGCAAAATTCATGAGGTCC	2	1X
53_LEFT	AGCAAAATGTTGGACTGAGACTGA	1	1X
53_RIGHT	AGCCTCATAAAACTCAGGTTCCC	1	1X
54_LEFT	TGAGTTAACAGGACACATGTTAGACA	2	1X
54_RIGHT	AACCAAAAACTTGTCCATTAGCACA	2	1X

55_LEFT	ACTCAACTTTACTTAGGAGGTATGAGCT	1	1X
55_RIGHT	GGTGTACTCTCCTATTTGTACTTTACTGT	1	1X
56_LEFT	ACCTAGACCACCACTTAACCGA	2	1X
56_RIGHT	ACACTATGCGAGCAGAAGGGTA	2	1X
57_LEFT	ATTCTACACTCCAGGGACCACC	1	1X
57_RIGHT	GTAATTGAGCAGGGTCGCCAAT	1	1X
58_LEFT	TGATTTGAGTGTTGTCAATGCCAGA	2	1X
58_RIGHT	CTTTTCTCCAAGCAGGGTTACGT	2	1X
59_LEFT	TCACGCATGATGTTTCATCTGCA	1	1X
59_RIGHT	AAGAGTCCTGTTACATTTTCAGCTTG	1	1X
60_LEFT	TGATAGAGACCTTTATGACAAGTTGCA	2	1X
60_RIGHT	GGTACCAACAGCTTCTCTAGTAGC	2	1X
61_LEFT	TGTTTATCACCCGCGAAGAAGC	1	1X
61_RIGHT	ATCACATAGACAACAGGTGCGC	1	1X
62_LEFT	GGCACATGGCTTTGAGTTGACA	2	1X
62_RIGHT	GTTGAACCTTTCTACAAGCCGC	2	1X
63_LEFT	TGTTAAGCGTGTTGACTGGACT	1	1X
63_RIGHT	ACAAACTGCCACCATCACAACC	1	1X
64_LEFT	TCGATAGATATCCTGCTAATTCCATTGT	2	7X
64_RIGHT	AGTCTTGTAAAAGTGTTCCAGAGGT	2	7X
65_LEFT	GCTGGCTTTAGCTTGTGGGTTT	1	1X
65_RIGHT	TGTCAGTCATAGAACAAACACCAATAGT	1	1X
66_LEFT	GGGTGTGGACATTGCTGCTAAT	2	7X
66_RIGHT	TCAATTTCCATTTGACTCCTGGGT	2	7X
67_LEFT	GTTGTCCAACAATTACCTGAAACTTACT	1	1X
67_RIGHT	CAACCTTAGAAACTACAGATAAATCTTGGG	1	1X
68_LEFT	ACAGGTTCATCTAAGTGTGTGTGT	2	1X
68_RIGHT	CTCCTTTATCAGAACCAGCACCA	2	1X
69_LEFT	TGTCGCAAAATATACTCAACTGTGTCA	1	1X
69_RIGHT	TCTTTATAGCCACGGAACCTCCA	1	1X
70_LEFT	ACAAAAGAAAATGACTCTAAAGAGGGTTT	2	7X
70_RIGHT	TGACCTTCTTTTAAAGACATAACAGCAG	2	7X
71_LEFT	ACAAATCCAATTCAGTTGTCTTCCTATTC	1	7X
71_RIGHT	TGGAAAAGAAAGGTAAGAACAAGTCCT	1	7X
72_LEFT	ACACGTGGTGTTTATTACCCTGAC	2	1X
72_RIGHT	ACTCTGAACTCACTTTCCATCCAAC	2	1X
73_LEFT	CAATTTTGTAATGATCCATTTTTGGGTGT	1	2.5X
73_RIGHT	CACCAGCTGTCCAACCTGAAGA	1	2.5X
74_LEFT	ACATCACTAGGTTTCAAACTTTACTTGC	2	7X
74_RIGHT	GCAACACAGTTGCTGATTCTCTTC	2	7X
75_LEFT	AGAGTCCAACCAACAGAATCTATTGT	1	7X
75_RIGHT	ACCACCAACCTTAGAATCAAGATTGT	1	7X
76_LEFT	AGGGCAAACTGGAAAGATTGCT	2	2.5X

76_LEFT_alt3	GGGCAAACTGGAAAGATTGCTGA	2	2.5X
76_RIGHT	ACACCTGTGCCTGTTAAACCAT	2	2.5X
76_RIGHT_alt0	ACCTGTGCCTGTTAAACCATTGA	2	2.5X
77_LEFT	CCAGCAACTGTTTGTGGACCTA	1	1X
77_RIGHT	CAGCCCCTATTAAACAGCCTGC	1	1X
78_LEFT	CAACTTACTCCTACTTGGCGTGT	2	1X
78_RIGHT	TGTGTACAAAAACTGCCATATTGCA	2	1X
79_LEFT	GTGGTGATTCAACTGAATGCAGC	1	1X
79_RIGHT	CATTTCATCTGTGAGCAAAGGTGG	1	1X
80_LEFT	TTGCCTTGGTGATATTGCTGCT	2	1X
80_RIGHT	TGGAGCTAAGTTGTTTAACAAGCG	2	1X
81_LEFT	GCACTTGGAAAACTTCAAGATGTGG	1	2.5X
81_RIGHT	GTGAAGTTCTTTTCTTGTGCAGGG	1	2.5X
82_LEFT	GGGCTATCATCTTATGTCCTTCCCT	2	1X
82_RIGHT	TGCCAGAGATGTCACCTAAATCAA	2	1X
83_LEFT	TCCTTTGCAACCTGAATTAGACTCA	1	2.5X
83_RIGHT	TTTGACTCCTTTGAGCACTGGC	1	2.5X
84_LEFT	TGCTGTAGTTGTCTCAAGGGCT	2	1X
84_RIGHT	AGGTGTGAGTAAACTGTTACAAACAAC	2	1X
85_LEFT	ACTAGCACTCTCCAAGGGTGTT	1	2.5X
85_RIGHT	ACACAGTCTTTTACTCCAGATTCCC	1	2.5X
86_LEFT	TCAGGTGATGGCACAACAAGTC	2	2.5X
86_RIGHT	ACGAAAGCAAGAAAAAGAAGTACGC	2	2.5X
87_LEFT	CGACTACTAGCGTGCCTTTGTA	1	1X
87_RIGHT	ACTAGGTTCCATTGTTCAAGGAGC	1	1X
88_LEFT	CCATGGCAGATTCCAACGGTAC	2	1X
88_RIGHT	TGGTCAGAATAGTGCCATGGAGT	2	1X
89_LEFT	GTACGCGTTCCATGTGGTCATT	1	2.5X
89_LEFT_alt2	CGCGTTCCATGTGGTCATTCAA	1	2.5X
89_RIGHT	ACCTGAAAGTCAACGAGATGAAACA	1	2.5X
89_RIGHT_alt4	ACGAGATGAAACATCTGTTGTCACT	1	2.5X
90_LEFT	ACACAGACCATTCCAGTAGCAGT	2	1X
90_RIGHT	TGAAATGGTGAATTGCCCTCGT	2	1X
91_LEFT	TCACTACCAAGAGTGTGTTAGAGGT	1	7X
91_RIGHT	TTCAAGTGAGAACCAAAAGATAATAAGCA	1	7X
92_LEFT	TTTGTGCTTTTTAGCCTTTCTGCT	2	1X
92_RIGHT	AGGTTCCTGGCAATTAATTGTAAAAGG	2	1X
93_LEFT	TGAGGCTGGTTCTAAATCACCCA	1	1X
93_RIGHT	AGGTCTTCCTTGCCATGTTGAG	1	1X
94_LEFT	GGCCCCAAGGTTTACCCAATAA	2	1X
94_RIGHT	TTTGGCAATGTTGTTCCTTGAGG	2	1X
95_LEFT	TGAGGGAGCCTTGAATACACCA	1	7X
95_RIGHT	CAGTACGTTTTTGCCGAGGCTT	1	7X

96_LEFT	GCCAACAACAACAAGGCCAAAC	2	1X
96_RIGHT	TAGGCTCTGTTGGTGGGAATGT	2	1X
97_LEFT	TGGATGACAAAGATCCAAATTTCAAAGA	1	7X
97_RIGHT	ACACACTGATTAAAGATTGCTATGTGAG	1	7X
98_LEFT	AACAATTGCAACAATCCATGAGCA	2	2.5X
98_RIGHT	TTCTCCTAAGAAGCTATTAAAATCACATGG	2	2.5X

Primer sequences are from the ARTICv3 primer set (Quick 2020).

[1] Relative primer concentration in each pool is indicated under Ratio.

Quick J, Loman NJ. hCoV-2019/nCoV-2019 Version 3 Amplicon Set. March 24, 2020. Available: https://artic.network/resources/ncov/ncov-amplicon-v3.pdf.

Table S3. SARS-CoV-2 primer binding site mutations found in samples (n=3,506) collected between January and February of 2021

Position[1]	Reference >Variant	Prevalence in the study cohort, %[2]	ARTICv3 Primer Name	Primer binding site mutation[3] (5' to 3')
1,578	T>C	1.3	nCoV-2019_6_LEFT	GGTGcTGTTGGAGAAGGTTCCG
2,258	G>A	2.7	nCoV-2019_7_RIGHT	TGCACAGGTGAtAATTTGTCCA
2,258	G>A	2.7	nCoV-2019_7_RIGHT_alt5	AGGTGAtAATTTGTCCACCGAC
4,683	C>T	1.0	nCoV-2019_15_RIGHT_alt3	ACTGTAaCTGGCACTTTGAGAGA
5,011	A>C	1.7	nCoV-2019_16_RIGHT	CACAACgTGCGTGTGGAGGTTA
5,869	C>T	2.5	nCoV-2019_20_LEFT	AtAAAGAAAACAGTTACACAACAACCA
6,730	C>T	1.0	nCoV-2019_23_LEFT	ACAACTACTAAtATAGTTACACGGTGT
8,264	G>T	1.7	nCoV-2019_28_LEFT	ACATAGAAGTTACTtGCGATAGTTGT
9,204	A>G	2.7	nCoV-2019_31_LEFT	TTCTGAGTACTGTAGGCACGGC
10,369	C>T	1.3	nCoV-2019_35_LEFT	TGTTCGtATTCAACCAGGACAG
10,741	C>T	2.3	nCoV-2019_35_RIGHT	ACTTCATAGCCACAAGGTTAAAaTCA
12,484	C>T	1.4	nCoV-2019_41_RIGHT	TGGTATaACAACCATTAGTTTGGCT
12,789	C>T	4.2	nCoV-2019_42_RIGHT	CCTACCTCCCTTTaTTGTGTTGT
13,019	C>T	2.3	nCoV-2019_44_LEFT	TGCCACAGTACGTtTACAAGCT
13,019	C>T	2.3	nCoV-2019_44_LEFT_alt3	CCACAGTACGTtTACAAGCTGG
16,500	A>C	1.3	nCoV-2019_54_RIGHT	AACCAAAAACgTGTCCATTAGCACA
21,364	C>T	1.9	nCoV-2019_71_LEFT	ACAAATtCAATTCAGTTGTCTTCCTATTC
22,018	G>T	17.7	nCoV-2019_72_RIGHT	ACTCTGAACTCACTTTCCATaCAAC
22,335	G>T	1.9	nCoV-2019_73_RIGHT	CACCAGCTGTCaAACCTGAAGA
23,191	C>T	1.4	nCoV-2019_76_RIGHT	ACACCTGTGCCTGTTAAACCAT
25,907	G>T	41.6	nCoV-2019_86_LEFT	TCAGtTGATGGCACAACAAGTC
26,305	C>G	1.3	nCoV-2019_86_RIGHT	ACGAAAGCAAcAAAAAGAAGTACGC
28,087	C>T	2.7	nCoV-2019_93_LEFT	TGAGGtTGGTTCTAAATCACCCA
29,362	C>T	17.9	nCoV-2019_96_RIGHT	TAGGCTCTGTTGGTGGaAATGT
29,377	T>A	1.5	nCoV-2019_96_RIGHT	TtGGCTCTGTTGGTGGGAATGT

[1] Variant position is shown against a SARS-CoV-2 reference genome 0MN908947.3.

[2] Shown only prevalence >1%

[3] Red lower case indicates mutation in the primer binding site.

Table S4. SARS-CoV-2 clade comparison between the high-throughput 2-step PCR workflow and a standard ARTIC v3 workflow

		Clade by ARTIC v3 method														
	Clade[1]	19B	20A	20B	20C	20G	20H (Beta, V2)	20I (Alpha, V1)	20J (Gamma, V3)	21A (Delta)	21C (Epsilon)	21D (Eta)	21F (Iota)	21G (Lambda)	21H	Total
Clade by 2-step PCR method	19B	3														3
	20A		53													53
	20B			105				1								106
	20C				98											98
	20G					175										175
	20H (Beta, V2)						7									7
	20I (Alpha, V1)							713								713
	20J (Gamma, V3)								44							44
	21A (Delta)									1						
	21C (Epsilon)										101					101
	21D (Eta)											10				10
	21F (Iota)												131			131
	21G (Lambda)													1		1
	21H														3	3
	Total	3	53	105	98	175	7	714	44		101	10	131	1	3	1446

[1] SARS-CoV-2 clades were assigned with Nextclade v1.3.0 (https://clades.nextstrain.org/).

Table S5. SARS-CoV-2 lineage comparison between the high-throughput 2-step PCR workflow and a standard ARTIC v3 workflow[1]

Lineage by ARTIC v3 method

Lineage by high-throughput 2-step method

Lineage*	A.2.5	AZ.3	B.1	B.1.1	B.1.1.207	B.1.1.222	B.1.1.306	B.1.1.316	B.1.1.318	B.1.1.345	B.1.1.348	B.1.1.434	B.1.1.519	B.1.1.7	B.1.110.3	B.1.189	B.1.2	B.1.214.2	B.1.232	B.1.234	B.1.239	B.1.243	B.1.243.1	B.1.265	B.1.298	B.1.311	B.1.351	B.1.36.29	B.1.404	B.1.427	R.1	Total
A.2.5	3																														3
AZ.3		3																													3
B.1			6																												6
B.1.1				2																											2
B.1.1.207					1																										1
B.1.1.222						8																									8
B.1.1.306							1																								1
B.1.1.316								2																							2
B.1.1.318									4																						4
B.1.1.345										1																					1
B.1.1.348											4																				4
B.1.1.434												2																			2
B.1.1.519													53	1																	54
B.1.1.7														702																	702
B.1.110.3															1																1
B.1.189																1															1
B.1.2																	153														153
B.1.214.2																		2													2
B.1.232																			1												1
B.1.234																				15											15
B.1.239																					1										1
B.1.243																						10									10
B.1.243.1																							2								2
B.1.265																								1							1
B.1.298																									1						1
B.1.311																										3					3
B.1.351																											7				7
B.1.36.29																												1			1
B.1.404																													1		1
B.1.427																														19	19
R.1																															22	22

Lineage*	B.1.429	B.1.429.1	B.1.475	B.1.517	B.1.525	B.1.526	B.1.539	B.1.561	B.1.568	B.1.575	B.1.588	B.1.595	B.1.596	B.1.604	B.1.609	B.1.617.2	B.1.621	B.1.623	B.1.625	B.1.628	B.1.634	B.1.635	B.1.637	C.37	N.5	P.1	P.1.10	P.1.2	P.2	Q.3	Q.4	Q.8	Total
B.1.429	80																																80
B.1.429.1		1																															1
B.1.475			1																														1
B.1.517				2																													2
B.1.525					10																												10
B.1.526						131																											131
B.1.539							1																										1
B.1.561								6																									6
B.1.568									3																								3
B.1.575	1									13																							14
B.1.588											3																						3
B.1.595												2																					2
B.1.596													24																				24
B.1.604														1																			1
B.1.609															1																		1
B.1.617.2																1																	1
B.1.621																	3																3
B.1.623																		9															9
B.1.625																			2														2
B.1.628																				3													3
B.1.634																					1												1
B.1.635																						1											1
B.1.637																							54										54
C.37																								1									1
N.5																									1								1
P.1																										29							29
P.1.10																											14						14
P.1.2																												1					1
P.2																													1				1
Q.3																														2			2
Q.4																															8		8
Q.8																																1	1
Total																																	1446

[1]Lineages were assigned with Pangoling v3.1.11 (https://pangolin.cog-uk.io/)

Table S6. SARS-CoV-2 primer binding site mutations causing amplicon dropout by a standard ARTIC v3 workflow

Clade	Number Sample	Failed Amplicon	Affected Primer	Primer Sequence[1] (5' to 3')	Position[2]	Reference	Variant
20H (Beta, V2)	7	ARTICv3-74	nCoV-2019_74_LEFT	ACATCACTAGGTTTCAAAC(tttacttgc)	22281	C	-TTTACTTGC
21A (Delta)	1	ARTICv3-72	nCoV-2019_72_RIGHT	ACTC(tgaact)CACTTTCCATCCAAC	22028	G	-AGTTCA
21C (Epsilon)	113	ARTICv3-72	nCoV-2019_72_RIGHT	ACTCTGAACTCACTTTCCATaCAAC	22018	G	T
21G (Lambda)	1	ARTICv3-97	nCoV-2019_97_LEFT	TGGATGACAAAGATCCAAATTTtAAAGA	29311	C	T
			nCoV-2019_97_RIGHT	ACACACTGATTAAAGATTaCTATGTGAG	29675	C	T

[1] Red lower case indicates mutation in the primer binding site; deleted nucleotides are shown in parentheses.

[2] Variant position is shown against a SARS-CoV-2 reference genome MN908947.3.

Table S7. Proportion of samples generating complete SARS-CoV-2 consensus sequence by the high-throughput workflow and a standard ARTIC v3 workflow

Clade	High-throughput workflow				ARTIC v3 workflow			
	Number complete	Number incomplete	Total assessed	Percent complete	Number complete	Number incomplete	Total assessed	Percent complete
20H (Beta, V2)	4	1	5	80.0	16	69	85	18.8
21A (Delta)	855	42	897	95.3	4	515	519	0.8
21C (Epsilon)	7	0	7	100.0	5	256	261	1.9
21G (Lambda)	15	3	18	83.3	1	29	30	3.3
Total	881	46	927	95.0	26	869	895	2.9

A different sample set was used between the two workflows.
The percent genome coverage was determined by counting the number of nucleotides meeting minimum coverage requirement divided by the total SARS-CoV-2 genome length excluding the 5’ (1-54nt) and 3’ (29,836–29,903nt) ends not covered by the amplicon panel.

Table S8. SARS-CoV-2 clade distribution of clinical cases analyzed between January and September 2021

Clade[1]	Number of cases	Percent
21A (Delta)	23,239	35.13
20I (Alpha V1)	19,022	28.76
20G	5,665	8.56
21C (Epsilon)	3,475	5.25
21F (Iota)	3,259	4.93
20C	3,103	4.69
20B	2,558	3.87
20J (Gamma V3)	2,540	3.84
20A	1,929	2.92
21H	683	1.03
20H (Beta V2)	156	0.24
19B	141	0.21
21G (Lambda)	140	0.21
21D (Eta)	131	0.2
20D	68	0.1
21B (Kappa)	25	0.04
20E (EU1)	12	0.02
Total	66,146	100

[1] SARS-CoV-2 clades were assigned with Nextclade v1.3.0 (https://clades.nextstrain.org/).
